# Machine learning models development for accurate multi-months ahead drought forecasting: Case study of the Great Lakes, North America

**DOI:** 10.1371/journal.pone.0290891

**Published:** 2023-10-31

**Authors:** Mohammed Majeed Hameed, Siti Fatin Mohd Razali, Wan Hanna Melini Wan Mohtar, Norinah Abd Rahman, Zaher Mundher Yaseen

**Affiliations:** 1 Department of Civil Engineering, Faculty of Engineering and Built Environment, Universiti Kebangsaan Malaysia (UKM), Bangi, Selangor, Malaysia; 2 Green Engineering and Net Zero Solution (GREENZ), Universiti Kebangsaan Malaysia (UKM), Bangi, Selangor, Malaysia; 3 Civil and Environmental Engineering Department, King Fahd University of Petroleum & Minerals, Dhahran, Saudi Arabia; 4 Interdisciplinary Research Center for Membranes and Water Security, King Fahd University of Petroleum & Minerals, Dhahran, Saudi Arabia; King Fahd University of Petroleum and Minerals, SAUDI ARABIA

## Abstract

The Great Lakes are critical freshwater sources, supporting millions of people, agriculture, and ecosystems. However, climate change has worsened droughts, leading to significant economic and social consequences. Accurate multi-month drought forecasting is, therefore, essential for effective water management and mitigating these impacts. This study introduces the Multivariate Standardized Lake Water Level Index (MSWI), a modified drought index that utilizes water level data collected from 1920 to 2020. Four hybrid models are developed: Support Vector Regression with Beluga whale optimization (SVR-BWO), Random Forest with Beluga whale optimization (RF-BWO), Extreme Learning Machine with Beluga whale optimization (ELM-BWO), and Regularized ELM with Beluga whale optimization (RELM-BWO). The models forecast droughts up to six months ahead for Lake Superior and Lake Michigan-Huron. The best-performing model is then selected to forecast droughts for the remaining three lakes, which have not experienced severe droughts in the past 50 years. The results show that incorporating the BWO improves the accuracy of all classical models, particularly in forecasting drought turning and critical points. Among the hybrid models, the RELM-BWO model achieves the highest level of accuracy, surpassing both classical and hybrid models by a significant margin (7.21 to 76.74%). Furthermore, Monte-Carlo simulation is employed to analyze uncertainties and ensure the reliability of the forecasts. Accordingly, the RELM-BWO model reliably forecasts droughts for all lakes, with a lead time ranging from 2 to 6 months. The study’s findings offer valuable insights for policymakers, water managers, and other stakeholders to better prepare drought mitigation strategies.

## 1. Introduction

Drought is a harmful and creeping climatic crisis that can occur in all climates and negatively influences ecosystems and human societies. Unlike rapidly developing natural disasters (e.g., hurricanes, floods, and storms), drought is a slow-developing phenomenon that poses a significant and enduring threat to human daily life, agriculture, ecology, and the economy [1, 2]. The lack of precipitation for a long time is the direct and leading cause of drought [3]. Since two decades ago, droughts have become increasingly common and severe due to climate change brought on by global warming, causing food and water crises in some regions worldwide [4–6]. Moreover, the occurrence of drought leads to various issues, including desertification, water scarcity, and forest fires, which have negative repercussions at both local and global scales [7–10]. Furthermore, drought that occurs in areas located in the North American continent and other countries has a great relationship with forest fires [11, 12]. Natural disasters that struck almost all world countries have severely affected enormous numbers of people all over the globe. Statistically analyzed data estimates that about 3.8 billion people worldwide have suffered from these catastrophes and that half of those have suffered due to droughts [13]. Also, the drought disaster has directly or indirectly killed 1.3 million people out of 3.5 million who died due to natural disasters.

Drought is typically categorized into four types: meteorological drought, agricultural drought, hydrological drought, and socio-economic drought [14–16]. The three physical drought categories (i.e., metrological, agricultural, and hydrological droughts) self-explanatorily describe the lack of water in the hydrological cycle. It can be said that drought is associated significantly with precipitation, and thus the onset of metrological drought begins when there is a rainfall deficit. Hydrological droughts often deal with the decrease in the river flow, groundwater level, dam reservoir storage, and lake water level due to long-term meteorological droughts [17]. In addition, persistent hydrological drought causes agricultural droughts. In agricultural drought, soil moisture is deficient, resulting in a reduction in crop yields.

Several drought indices have been developed since the 1960s for monitoring and forecasting droughts based on individual or multiple meteorological or hydrological variables (e.g., rainfall, evapotranspiration, streamflow, groundwater level, and runoff) [18]. It is worth mentioning that among all drought indexes, the "standardized precipitation index (SPI)", proposed by [19], is broadly used to quantify drought across the world and receives considerable attention from researchers [5, 20, 21]. Based on SPI principles, several hydrological parameters have been derived and widely used to assess and monitor droughts, such as standardized streamflow index (SSI) [22, 23], standardized hydrological drought index (SHDI) [24], standardized groundwater level index (SGI) [25], and standardized runoff index (SRI) [26, 27]. These indices’ popularity and widespread use can be attributed to their ability to effectively evaluate and track drought conditions across diverse timeframes and geographic locations while requiring minimal data inputs and maintaining strong comparability in time and space [28, 29].

Since drought is a manifestation of climate variability events, scholars have developed drought forecasting models to minimize the adverse effects of drought on agriculture, water resource systems, socio-economic aspects, and hydropower generation. Notably, climate variability events refer to natural fluctuations in climate patterns that can affect temperature, precipitation, and other climate factors over time. Climate change can intensify the impacts of drought, leading to more frequent occurrences of water scarcity [30, 31]. This poses significant challenges for ecosystems and communities that depend on reliable access to water resources. Thus, drought forecasting is substantial and necessary to make effective decisions in managing water resources, executing drought mitigation strategies, and establishing robust early warning systems [32]. Therefore, selecting an appropriate model is the most crucial factor in drought forecasting after identifying the drought type. The forecast of droughts is frequently implemented using physical and data-driven models. Conceptual and physical models that necessitate substantial data about a catchment or basin process, resulting in complicated predicting models [24]. However, data-driven models are more popular because they are flexible, require less data, and do not consider the process of a studied catchment. In recent decades, different machine learning (ML) models and statistical approaches were intensively utilized, such as autoregressive integrated moving average [33, 34], support vector regression [35–37], adaptive neuro-fuzzy inference system [38–40], artificial neural network (ANN) [41–43], deep learning models [44, 45], and assembling based approaches like random forest [46, 47] for forecasting drought over the world. Some researchers have developed hybrid models based on nature-inspired algorithms that are effective and adaptable in forecasting drought. The meta-heuristic algorithms can help produce models with high prediction accuracy by training or optimizing the parameters of ML models, which substantially affects the model performance [48, 49]. Notably, fourteen meta-algorithms have been intensively used to improve the ML model’s performance in drought forecasting [24, 50–54].

Enhancing the structure of ML-based models in drought forecasting has aroused the interest of researchers in the last few years. Some scholars tackled fundamental issues of some ML models, such as ANN. Despite being a popular approach among ML-based models in drought sectors, this model encounters difficulties related to generalization, complexity, and learning speed, along with the challenge of becoming stuck in a local minimum. Thus, an extreme learning machine (ELM) was introduced to address the drawbacks of ANN (i.e., complexity, slow convergence, and overfitting) and then used widely to solve engineering problems and drought forecasting. ELM has several advantages compared to ANN, such as better generalization, fast learning, simpler structure, and superior performance [55, 56]. Accordingly, ELM has been applied for drought forecasting in different climate regions and obtained promising results [1, 30, 57, 58]. Nevertheless, it is possible to adjust the model structure to enhance its performance and stability by using regularization parameters and nature-inspired algorithms to train the model effectively.

Researchers have not paid enough attention to monitoring and forecasting hydrological drought, despite its importance in managing water resources [59]. The main objective of this study is to evaluate and forecast hydrological drought in the Great Lakes, situated in North America, which contains over one-fifth of the world’s fresh water. The Great Lakes are an essential source of fresh water for people, and droughts in these water bodies can significantly impact water availability and the ecosystem. Notably, the recent droughts in the Great Lakes have been mainly attributed to climate change-induced elevated water temperatures, resulting in increased evaporation due to a substantial decline in winter ice cover [60]. This study used Multivariate Standardized Lake Water Level Index (MSWI) to assess the hydrological drought over the Grate Lakes. MSWI can simultaneously display the drought condition from the standpoints of many time scales. To forecast hydrological drought for different lead-time horizons (from one to six months ahead), four hybrid models were developed by combining regularized extreme learning machine (RELM), RF, ELM, and SVR with the Beluga whale optimization (BWO) algorithm. The BWO algorithm is a novel nature-inspired algorithm with robust and efficient performance in solving engineering problems. Moreover, it displays excellent and superior results when tested against fifteen meta-heuristic algorithms in solving different engineering problems [61]. The hybrid models were compared and validated against established standard models (RF, ELM, and SVR).

Previous drought works have suggested using standard performance metrics such as correlation of determinations (*R*^*2*^), root mean square error (RMSE), and others to choose the best prediction models [62, 63]. However, drought time series data, particularly at higher time scales, include a significantly high auto-correlation [64–66]. This can result in very high *R*^*2*^ values and lower forecasting errors, rendering such metrics less effective in selecting the best models. Accordingly, this paper’s proposed advanced evaluation method focuses on accurately detecting the turning points in drought data and evaluating the model’s ability to capture them effectively. Finally, uncertainty analysis was used to evaluate the forecasting accuracy and select which period lead times can be reliably forecasted.

## 2. Methodological overview

### 2.1 Random forest (RF)

The principle of an ensemble and bagging learning technique served as the foundation for creating the RF. The decision tree approach utilizing the bagging methodology is a crucial factor in the effectiveness of this model in solving engineering problems, especially those related to regression [67]. In RF, nodes are nominated randomly based on the most significant input features, enhancing the learning, forecasting, and maintaining reliability to improve the generalization capacity. The following procedures are considered to establish the RF model [57]:

- From training data observations, select random *i* data samples bootstrap sample.
- Establish the decision tree related to the data selected (*i*) from the above step.
- Select the n-trees (n_trees_) that require to be built.
- Repeat the steps from one to three.
- Forecast MSWI by accumulating the aggregative forecasts of n_trees_.

**Fig 1:**
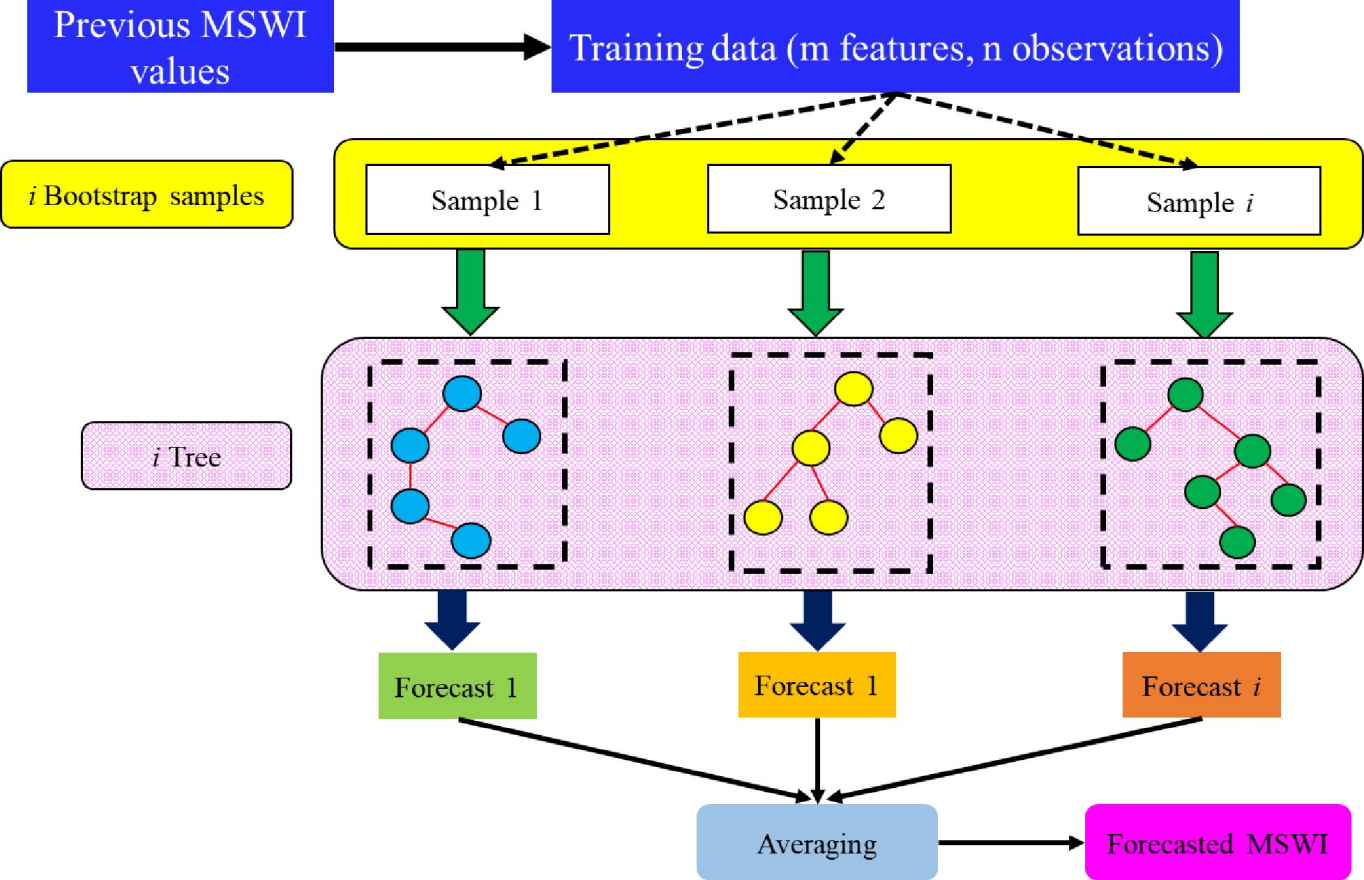
Schematic of RF model.

The RF model’s capacity has gained a good reputation in solving hydrological and environmental problems and also conducted in drought forecasting [46, 68–71]. Fig 1 provides the random forest model’s flowchart.

### 2.2 Support vector regression (SVR)

SVR is a supervisor learning machine used widely in water resource applications and drought forecasting. The main idea behind SVR is to map the input vectors to higher feature dimensional spaces through a nonlinear mapping and then conduct a regression model based on the calculated feature spaces. Even with limited observations from the training datasets, the approach can effectively find an excellent solution to nonlinear problems [48, 72]. The structural risk minimization (SRM) concept, which SVM employs, seeks to minimize the upper and lower bounds of the generalization error, which is the sum of the training error and a confidence level [73, 74]. This concept is distinct from the more common empirical risk minimization (ERM) method, which focuses only on reducing the total error generated during the training stage.

Assuming the *MSWI*_*i*_ in the datapoints is the actual normalized value of the *i*^*th*^ sample while the input parameter of that target is *X*_*i*_. The sample set accordingly can be derived as {(*Xi*, *Yi*)}_*i*=1_^*N*^ where the *N* is the total samples, SVR uses the form in the following Equation to approximate the function. Finally, the term *Y*_*i*_ is this case is *MSWI*_*i*_.


MSWI=f(X)=W.∅(X)+b (1)

The ∅(*X*) is the high-dimensional space. Estimating coefficients (*W* and *b)* requires using a regularized risk function, represented by Eq (2).


$$Minimize:\;\frac{1}{2}{\left\|W\right\|}^{2}+C\frac{1}{N}\sum_{i=1}^{N}L_{\varepsilon }(Y_{i},f(X_{i}))$$ (2)

$$L_{\varepsilon }({MSWI}_{i},f(X_{i}))=\left\{ \begin{array}{l} \;0,\;|Y_{i}-f(X_{i})|\leq \varepsilon \\ |{MSWI}_{i}-f(X_{i})|\;Others \end{array}\right.$$ (3)

The regularized term in the above equations is ‖*W*‖^2^, and *C* to calculate the trade-off between training error and model flatness. In Eq (2), the second term represents the empirical error determined by the intensity loss function (Eq 3). The loss function equals zero if the anticipated value corresponds with the tube (a region around the predicted regression line). Conversely, the loss is a value representing the difference between the tube residual of the tube (*ε*) and the projected value [75]. The Equation above is converted into the principal objective function represented by Eq 4 to estimate the coefficients (*W* and *b*).


$$minmize\;({W,\xi }_{1},\xi_{1}^{*},b)\frac{1}{2}∥W{∥}^{2}+C\sum_{i=1}^{n}(\xi_{1}+\xi_{1}^{*})$$

$$Subject\;to:\;\left\{ \begin{array}{c} {MSWI}_{i}-W.\emptyset (x_{i})-b\leq \varepsilon +\xi_{1},\\ W.\emptyset (x_{i})+b\leq \varepsilon +\xi_{1}^{*},\;i=1,2,\ldots ,N\\ \xi_{1},\xi_{1}^{*}\geq 0 \end{array}\right.$$ (4)

In the Equation above the slack variables are *ξ*_1_, and $\xi_{1}^{*}$. Eq (5) is expressed as follows once the kernel function *K(X*_*i*_, *X*_*j*_*)* is introduced;

$$Minimize(\alpha_{i},\alpha_{i}^{*}):\frac{1}{2}\sum_{i=1}^{N}\sum_{j=1}^{N}(\alpha_{i}-\alpha_{i}^{*})(\alpha_{j}-\alpha_{j}^{*}).K(X_{i},X_{j})-\varepsilon \sum_{i=1}^{N}\left(\alpha_{i}-\alpha_{i}^{*}\right)+\sum_{i=1}^{N}{MSWI}_{i}(\alpha_{i}-\alpha_{i}^{*})$$

$$Subject\;to:\left\{ \begin{array}{c} \sum_{i=1}^{N}\left(\alpha_{i}-\alpha_{i}^{*}\right)=0\\ \alpha_{i},\alpha_{i}^{*}\in [0,C] \end{array}\right.$$ (5)

In the above Equation, the *α*_*i*_, and $\alpha_{i}^{*}$ are the Lagrange multipliers while the *i*, *j* are different samples that are used in the training dataset. Accordingly, Eq (3) can be expressed as below:

$$MSWI=f(X)=\sum_{i=1}^{N}\left(\alpha_{i}-\alpha_{i}^{*}\right)K(X_{i},X_{j})+b$$ (6)

Notably, the Gaussian kernel density function is used in this study [76].

### 2.3 Extreme learning machine (ELM)

**Fig 2:**
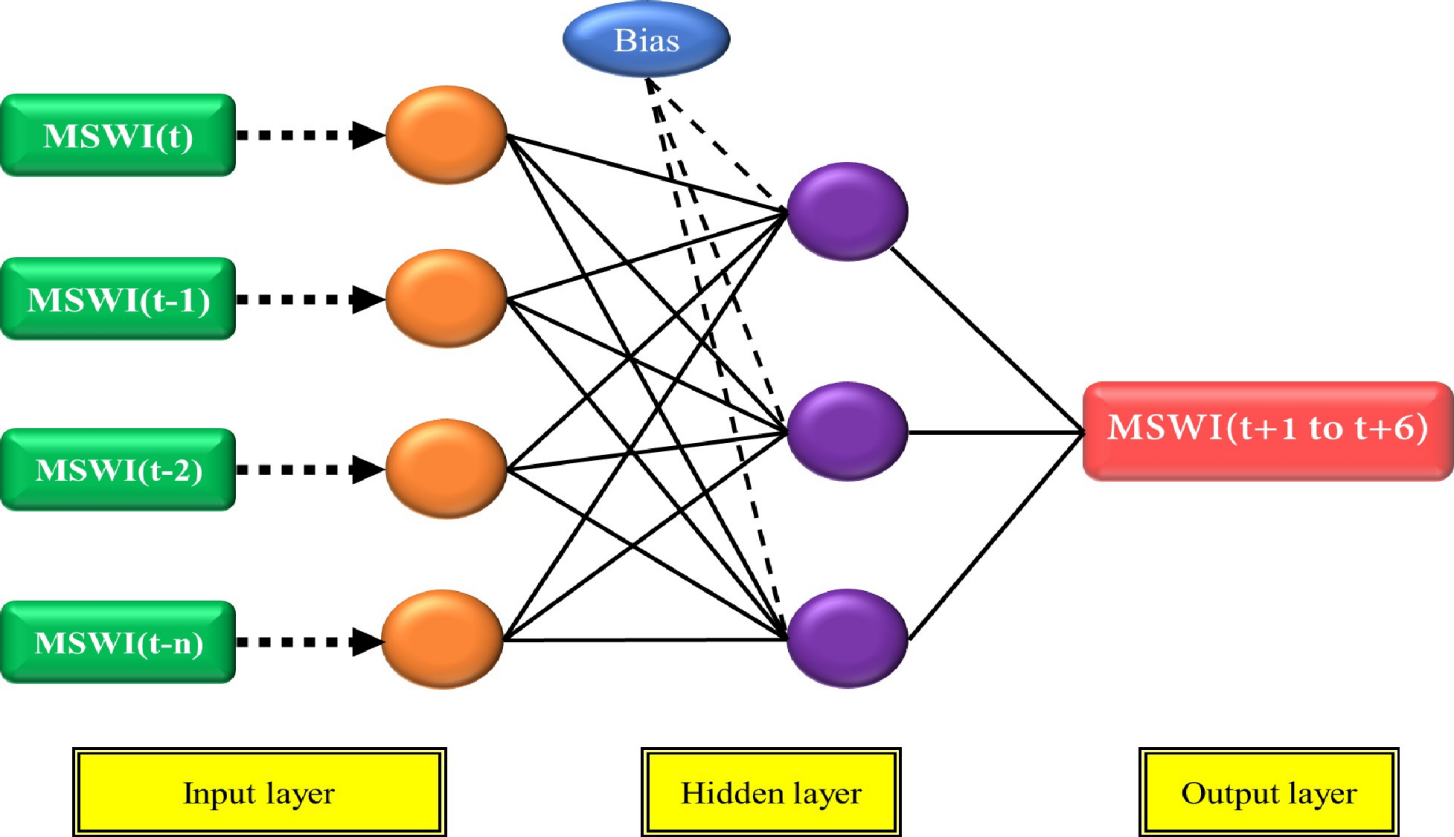
ELM model for forecasting MSWI.

ELM is a cutting-edge algorithm invented in 2006 to train the single-layer feedforward neural network. ELM’s inventor affirmed that this algorithm could replace the conventional algorithm (i.e., backpropagation algorithms) to train ANN due to its quick learning and superior generalization [77]. Fig 2 shows the general schematic of the ELM model. The construction of the ELM model can be expressed mathematically as follows:

$$f(x)=\sum_{i=1}^{M}\theta_{i}h_{i}(x_{i})=h(x)\theta$$ (7)

where *θ*_*i*_ = [*θ*_1_, *θ*_2_, *I*, *θ*_*M*_]^*T*^ is the output layer weight value. These values are significant because they connect the *m* output nodes in the hidden layer of *M* nodes. The nonlinear ELM feature mapping represents as *h*_*i*_(*x*_*i*_), *h*_*i*_(*x*_*i*_) = [*h*_*i*_(*x*), *h*_2_(*xI*…,*h*_*m*_(*x*)]^*T*^. The final ELM output or forecast is *f*(*x*). The *x*_*i*_ is the independent variable, and *h*_*i*_*(x)* is the output magnitude of the *i*^*th*^ hidden note output. It is possible in the ELM model that hidden nodes do not have unique output functions. It can be said that each hidden neuron may have a different output function. However, *H*_*i*_(*x*_*i*_) in practical problems can be expressed as:

$$H_{i}(x_{i})=G(a_{i},b_{i},x),a_{i}\in R^{d},b_{i}\in R$$ (8)

Parameters *a* and *b* are the hidden node parameters (weights and bias values), *x*_*i*_ is the input variable, *R* refers to the set of real number values, *R*^*d*^ is the d-dimensional set of real numbers, and *G* is the nonlinear transfer function. The transfer function is essential in the ELM model because it is responsible for mapping input data to a complex and higher dimensional space; hence, the model can find a satisfactory solution for complex engineering problems [78]. ELM algorithm through training ANN is based on two essential phases, the first stage is random features mapping, and the second one is called linear parameters solving. ELM uses several nonlinear activation functions to convert the inputs (i.e., variables) into feature space by randomly adjusting weights and bias values of t’e ELM’s hidden layer. Accordingly, the hidden layer parameters (*a*, *b*) are assigned randomly. The other significant stage in ELM is to calculate the weight values (*θ*) which connect the hidden layer of the ELM model with the output layer. These wights are linearly calculated using least square error sense by minimizing the approximations error:

$$\underset{\theta }{min}\|H\theta -Y\|$$ (9)

The term *Y* is this case is *MSWI* (training data) and the *H* matrix depends on input variables, and hidden layer parameters in the above formula can be calculated below [79]:

$$H=\left[\begin{array}{c} h(x_{1})\\ \vdots \\ h(x_{N}) \end{array}\right]=\left[\begin{array}{ccc} G(a_{1}x_{1}+b_{1}) & \cdots & G(a_{M}x_{1}+b_{M})\\ \vdots & \ddots & \vdots \\ G(a_{1}x_{N}+b_{1}) & \cdots & G(a_{M}x_{N}+b_{M}) \end{array}\right]$$ (10)

The *Y* is the training data response, which can be expressed as:

$$Y=\left[\begin{array}{c} {y_{1}}^{T}\\ \vdots \\ {y_{1}}^{T} \end{array}\right]=\left[\begin{array}{ccc} t_{11} & \cdots & t_{1M}\\ \vdots & \ddots & \vdots \\ t_{N1} & \cdots & t_{NM} \end{array}\right]$$ (11)

The ‖.‖ is the Frobenius norm, and thus, the calculated output weights |(θ*) can be determined as follow:

$$\theta^{*}=H^{+}Y$$ (12)

In the above mathematical expression, the *H*^+^ is the Moore–Penrose generalized inverse of the matrix of *H*. ELM differs from conventional neural networks in that there is no requirement to fine-tune every parameter of the model, including the weights and biases of the hidden layer. After random selection of the hidden layer weight and bias values, the ELM can be considered a linear system. Then, the weight values that connect the hidden layer with the output layer of ELM are systematically determined by the generalized inverse procedure of the hidden layer output matrices. As a result, the ELM model is several times quicker than classical algorithms used for training feedforward networks.

### 2.4 Regularized extreme learning machine

As discussed in the previous section, the only output weight matrix of the ELM model is calculated from the training procedure. The training error should decrease to a minimum value to obtain these weights. However, minimizing only the training error might reduce the generalization capacity of the developed model when estimating samples that were not included in training data points [80]. Thus, the classical ELM solutions would have some serious weaknesses. The first problem is that ELM, based on the empirical risk minimization (ERM) concept, frequently leads to over-fitting models [81]. Due to its direct calculation of the lowest norm least-squares solutions, ELM has a poor control capability, which is regarded as the second disadvantage. Another drawback is that it might lead to less reliable estimates, for example, if data includes outlier values or heteroskedasticity.

The highest level of generalizability is achieved in some advanced models when the weights’ norm values and forecasting training -error rate are minimized simultaneously [82]. Accordingly, this paper suggested RELM based on the structural risk minimization (SRM) concept of statistical learning theory to address mentioned disadvantages [83]. The uniqueness of this characteristic in the RELM model is attributed to its regularization parameter (*γ*). It is important to mention that the loss function for the ELM model is modified in this work using *γ* parameter to reduce both the training error and the weight values norm simultaneously. The mathematical expression of the cost function for RELM model can be illustrated below:

$$E_{RELM}=\underset{\theta }{Min}\;\gamma {{\left\|Y-H\theta \right\|}_{2}}^{2}+{{\left\|\theta \right\|}_{2}}^{2}$$ (13)

Subject to error *ε*:

ε=Y−Hθ (14)

Thus, the relation can be expressed as the following mathematical expression:

$$E_{RELM}=\underset{\theta }{Min}\;\gamma {{\left\|\varepsilon \right\|}_{2}}^{2}+{{\left\|\theta \right\|}_{2}}^{2}$$ (15)

The Lagrangian Equation is defined as the follow to calculate the optimum solution:

$$L(\theta ,\varepsilon ,\lambda )=\gamma {{\left\|\varepsilon \right\|}_{2}}^{2}+{{\left\|\theta \right\|}_{2}}^{2}+\lambda^{T}(Y-H\theta -\varepsilon )$$ (16)

$$\left\{ \begin{array}{c} \frac{\partial L}{\partial \theta }=0\Rightarrow 2\theta -H^{T}\lambda =0\\ \frac{\partial L}{\partial \varepsilon }=0\Rightarrow 2\theta -H^{T}\lambda =0\\ \frac{\partial L}{\partial \lambda }=0\Rightarrow Y-H\theta =0 \end{array}\right.$$ (17)

In the above equations, the Lagrangian multiplier matrix is *λ*, and *ε* is the training error, *ε* = [*ε*_1_, *ε*_2_,…*ε*_*N*_]^*T*^. The N is the total number of training samples. According to the previous equations, the optimal weights of the RELM output layer are computed as the following Equation:

$$\hat{\theta }={\left(H^{T}H\right)}^{-1}*\frac{I}{\gamma }H^{T}Y$$ (18)

The term *I* in the above Equation is called the unit matrix. The formula of calculated $\hat{\theta }$ vector is suitable when the hidden neurons are more than the training samples. Otherwise, the following Equation can be used to find the weights.


$$\hat{\theta }={H^{T}({HH}^{T}+\frac{I}{\gamma })}^{-1}*Y$$ (19)

### 2.5 Beluga whale algorithm (BWO)

BWO is a sophisticated metaheuristic algorithm invented in 2022 to solve engineering and optimization problems [61]. The algorithm was employed to improve the efficiency of drought forecasting by training the RELM model. BWO simulates the behaviour of beluga whales that live in regular groups, searching for and catching prey in the sea [84]. The mathematical model of BWO depends on three essential stages: exploration, exploitation, and whale fall. The first stage (exploration phase) in the mathematical model simulates the swimming behaviour of the Beluga whale, while the exploitation stage is inspired by preying behaviour of these animals. The third stage is inspired by the whale’s fall into the depths of the sea, or it was hunted by people or predators such as polar bears; hence it’s called "whale fall". It is worth noting that during the optimization process, the whale fall stage is executed in each iteration after the exploration and exploitation stages are completed. The main steps needed to implement this algorithm can be summarized as follows:

- Initialization: input the algorithm parameters, such as the maximum number of iterations (*T*_*max*_) and population size (*n*). The initial position of each population size (beluga whales) is randomly assigned in the space search. Besides, the fitness values are computed according to the given objective function.
- Extraction and exploration phases update: according to the balance factor (*Bf*) value, each beluga whale is appointed to be involved in the exploration or exploitation stage. If the *Bf* value exceeds 0.5, that means the Beluga whale is entering into the exploration phase, and thus its position will be updated according to the following Equation:
$$\left\{\begin{array}{c} {x_{i,j}}^{T+1}={x_{i,p_{j}}}^{T}+({x_{r,p_{1}}}^{T}-{x_{i,p_{j}}}^{T})+(1+r_{1})\sin\left(2\pi r_{2}\right),\;j=even\\ {x_{i,j}}^{T+1}={x_{i,p_{j}}}^{T}+({x_{r,p_{1}}}^{T}-{x_{i,p_{j}}}^{T})+(1+r_{1})\cos\left(2\pi r_{2}\right),\;j=odd \end{array}\right\}$$ (20)
  The current iteration in the evacuation is *T* while the new position of *i*th whale on *j*th dimensional space is *x*_*i*,*j*_^*T*+1^. The symbols *r*_1_, and *r*_2_ are random numbers between (0, 1). ${x_{i,p_{j}}}^{T}$ is the position of the *i*th beluga whale on *p*_*j*_ dimension.
  Otherwise, when the *Bf* value is less than 0.5, the updating mechanism is controlled by the exploitation stage, and eventually, the position of each whale is updated using Eq 21. After that, the fitness values of new positions are determined and sorted to achieve the best outcome in the current iteration.
$${X_{i}}^{T+1}=r_{3}{X_{best}}^{T}-r_{4}{X_{i}}^{T}+C_{1}.L_{F}.({X_{r}}^{T}-{X_{i}}^{T})$$ (21)
  *X*_*i*_^*T*^ and *X*_*r*_^*T*^ are current positions for the *i*th beluga whale and a random beluga whale, respectively. The two terms in the Equation (r_3_ and r_4_) are random numbers between (0, 1) while *C*_1_ is a constant number that depends on *r*_4_ current and maximum iteration (*T* and *T*_*max*_); $C_{1}=2r_{4}(1-\frac{T}{T_{max}})$. Finally, the *L*_*F*_ is the Levy flight function [85].
$$L_{F}=0.05\times \frac{\mu \times \sigma }{{\left|v\right|}^{\frac{1}{\beta }}}$$ (22)
$$\sigma ={\left(\frac{\Gamma (1+\beta )\times sin(\pi \beta /2)}{\Gamma ((1+\beta )/2)\times \beta \times 2^{(1-\beta )/2}}\right)}^{\frac{1}{\beta }}$$ (23)
  *β* is the default constant (1.5), while *μ*, and *v* are normally distributed random numbers.
- Whale fall phases update: The probability of whale fall (*W*_*f*_) is determined in each iteration, considering that some beluga whales may fall into the deep sea or die. Consequently, it is essential in such cases to update the position of a beluga whale.
$${X^{T+1}}_{i}=r_{5}{X_{i}}^{T}-r_{6}{X_{r}}^{T}+r_{7}X_{step}$$ (24)
  In the above equations, the *r*_5_, *r*_6_, and *r*_7_ are assigned randomly between one and zero. Besides, *X*_*step*_ is the step size of a whale fall.
$$X_{step}=(u_{b}-l_{b})\times exp(-C_{2}T/T_{max})$$ (25)
  In the above Equation, the *u*_*b*_, and *l*_*b*_ are upper and lower bond variables. The *C*_2_ is a step factor that depends mainly on *W*_*f*_.
$$C_{2}=2W_{f}\times n$$ (26)
  Finally, the probability of whale fall can be calculated using the following formula.
$$W_{f}=0.1-0.05\;T/T_{max}$$ (27)
- Terminating condition check: If the current iteration exceeds the maximum number of iterations, the BWO algorithm terminates. Otherwise, repeat steps ii to iii.

### 2.6 Model development

Multiple ML-based models have been created to forecast droughts in the Great Lakes region with the ability to forecast several months in advance. This study integrated a modified version of ELM called RELM with BWO to create a hybrid model known as RELM-BWO. The performance of this model was compared to classical models such as ELM, SVR, and RF. The RELM-BWO model was also validated against other hybrid models that combined classical models with BWO, namely SVR-BWO, RF-BWO, and ELM-BWO. All models were trained using past values (5-month lag) to forecast MSWI for several time steps ranging from 1 to 6 months ahead. In this study, time series drought data is divided into a training set (75% of the data from December 1921 to January 1997) and a testing set (data points from February 1997 to December 2021). This division ratio balances the amount of data for training and testing in machine learning models, resulting in good results [86, 87]. The method aims to train the model on historical data and assess its predictive efficiency on the remaining portion. This approach provides insights into the accuracy of future forecasts for drought data using historical information and is considered more challenging than random data division in time series analysis.

**Fig 3:**
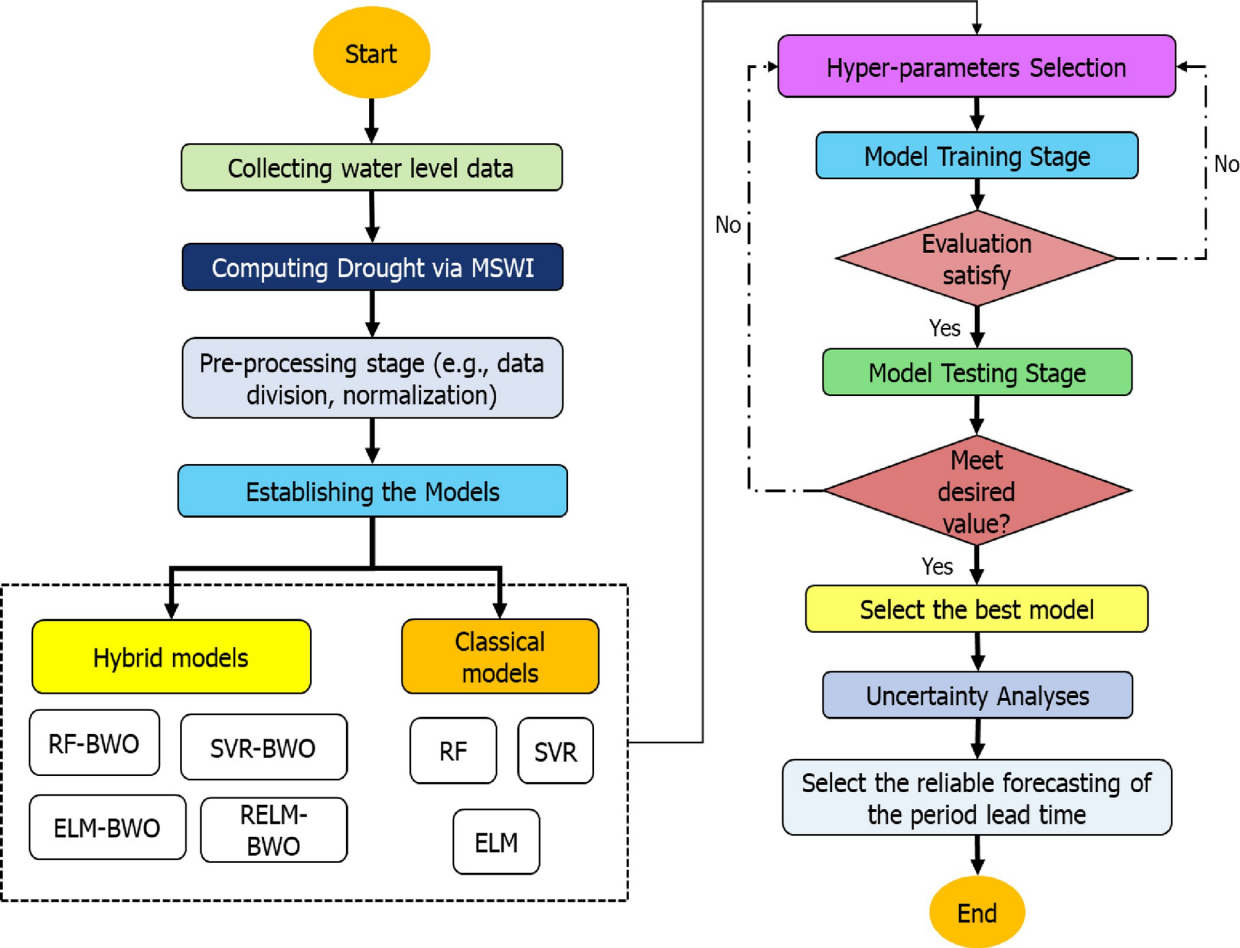
Flow chart of model development.

Once the input selection was determined, all input vectors and their corresponding targets were normalized to a range between 0 and 1. For the hybrid models, the primary objective of BWO was to optimize the hyperparameters of the classical models, as they greatly influence the accuracy of the model’s predictions. The objective function used was the root mean square error, and BWO aimed to minimize this function by selecting the best hyperparameters for each model. In the case of RELM-BWO, BWO was employed to determine the optimal value of the regularization parameter (*γ*) and the weight and bias values of the input layer. The remaining parameters for the other models calculated by BWO are listed in Table 1. All the models were implemented using MATLAB 2020a, and the primary process can be observed in Fig 3.

**Table 1:** Hyperparameters of the applied models.

Algorithm/ Model	Parameter
**RF**	Number of trees ranges from 26 to 70, while the leaf ∈ [3,8]
**SVR**	Kernel Scale range from 0.2441 to o.91061, and Box Constrain range from 0.1449 to 0.9554.
**ELM**	Weights range from -1 to 1, Hidden Layer Nodes are 7.
**BWO**	The maximum iteration is 100, and the number population is 50.
**RELM-BWO**	Weights range from -1 to 1, Hidden Layer Nodes are 7. BWO applied to compute hidden layer’s weights, biases values, and the Regularization parameter (*γ*)
**SVR-BWO**	BWO applied to compute Kernel Scale, Box Constrain, and Epsilon
**RF-BWO**	BWO applied to determine Number of trees, and leaf numbers.
**ELM-BWO**	Weights range from -1 to 1, Hidden Layer Nodes are 7. BWO applied to compute the hidden layer’s weights, and biases values,

### 2.7 Statistical parameters

The forecasting results were evaluated to identify the best forecasting model. Several statistical indicators have been used to assess the forecasting results, such as Mean absolute error (*MAE*), Root means square error (*RMSE*), Relative error (*RE*%), and Correlation of determination (*R*^*2*^). *MAE* is used widely to assess regression models in hydrological studies. This criterion measures the mean absolute forecasting error. *RMSE* is one of the most famous indices used to evaluate comparable models. *RMSE* measures the mean square forecasting error and has a higher value than *MAE*. Besides, *RE*% is the absolute error divided by the actual data value, while *R*^*2*^ is a measurement used to explain how much variability of the forecasted values can be caused by its relationship to the measured data. The mathematical expression of the statistical metrics can be seen as following [76]:

$$MAE=\frac{1}{n}\sum_{i=1}^{n}\left|{MSWI}_{{obs}_{i}}-{MSWI}_{{pred}_{i}}\right|$$ (28)

$$RMSE=\sqrt{\frac{1}{n}\sum_{i=1}^{N}{\left({MSWI}_{{obs}_{i}}-{MSWI}_{{pred}_{i}}\right)}^{2}}$$ (29)

$$RE\%=\frac{{MSWI}_{{obs}_{i}}-{MSWI}_{{pred}_{i}}}{{MSWI}_{{obs}_{i}}}\times 100\%$$ (30)

$$R^{2}=1-\frac{\sum_{i=1}^{n}({MSWI}_{{obs}_{i}}-{MSWI}_{{pred}_{i}}{)}^{2}}{\sum_{i=1}^{n}{\left({MSWI}_{{pred}_{i}}-\bar{{MSWI}_{pred}}\right)}^{2}}$$ (31)

The best forecasting models provide the possible lower value of *RMSE*, *RE%*, and the highest *R*^*2*^. In the above formulas, the *n* is the total number of samples, ${MSWI}_{{obs}_{i}}$ is the observed drought value, ${MSWI}_{{pred}_{i}}$ is the forecasted values, and $\bar{{MSWI}_{pred}}$ is the mean forecasted value.

### 2.8 Uncertainty analysis

The forecasting accuracy decreases when the lead time horizon increases [59, 88]; hence, adopting an efficient norm to assess forecasting efficiency is crucial. Since it is difficult to evaluate the reliability of the ML-based models via classical performance measures (i.e., *RMSE*, *R*^*2*^, etc.) in forecasting the drought at longer lead time scales, UN is used because it is an efficient tool to examine the dependability of forecasting results. Based on the concept of Monte Carlo Simulation, the UN of the adopted model is quantified. This study’s consideration of input parameter uncertainty relates to the accuracy and representativeness of the input data used to make forecasts [89]. The applied approach comprises producing random input data that matches the empirical probability distribution utilized to represent the input parameter, executing the forecasting model, and generating output from the data obtained [90]. The current study depends on randomly resampling the input data set without replacement 100000 times while maintaining the same ratio rate between the training and testing sets [91, 92]. In other words, for each observation, 100000 data points are generated from their corresponding empirical probability distribution and then fed to the applied model to generate the responses. To calculate the 95% confidence intervals for each response, the 2.5^*th*^(*X*_*L*_) and 97.5^*th*^(*X*_*U*_) percentiles of the cumulative distribution comprising 100000 datapoints are determined. The validity of the final drought model is assessed by calculating its robustness measure, which is determined by the proportion of observed drought values that fall within the 95% confidence interval. A higher ratio indicates greater validity, while a lower ratio indicates the opposite. The Equation for the 95% prediction uncertainty (95*PPU*) is provided below.


$$Bracketted\;by\;95\;PPU=\frac{1}{n}count(m|{X^{i}}_{L}\leq {X^{i}}_{Obs}\leq {X^{l}}_{U})\times 100$$ (32)

In the Eq 32, the term 95*PPU* denotes 95% predicted uncertainty; *n* is the total number of drought data records; *i* is the current month that can be changed from 1 to m; *X*^*i*^_*L*_, and *X*^*i*^_*U*_ are lower and upper bands of uncertainty, and *X*^*i*^_*Obs*_ is the observed data of ith month.

Additionally, the average width of the confidence interval is calculated using the *d*−*factor*, which can be expressed through the following Equation:

$$d-factor=\frac{\bar{d_{x}}}{\bar{\sigma_{x}}}$$ (33)

$$\bar{d_{x}}=\frac{1}{n}\sum_{i=1}^{n}\left({X_{U}}^{i}-{X_{L}}^{i}\right)$$ (34)

The $\bar{\sigma_{x}}$ is the standard deviation of the observed data while the average distance between the upper band (97.5^th^ percentile) and the lower band (2.5^th^ percentile). The higher value of the *d-factor* represents a higher degree of uncertainty. Theoretically, the *d-factor* is zero, but it normally cannot be attained due to the model uncertainty. Thus, the ideal value of the *d-factor* is thus considered to be smaller than one [93].

## 3. Case study and data description

**Fig 4:**
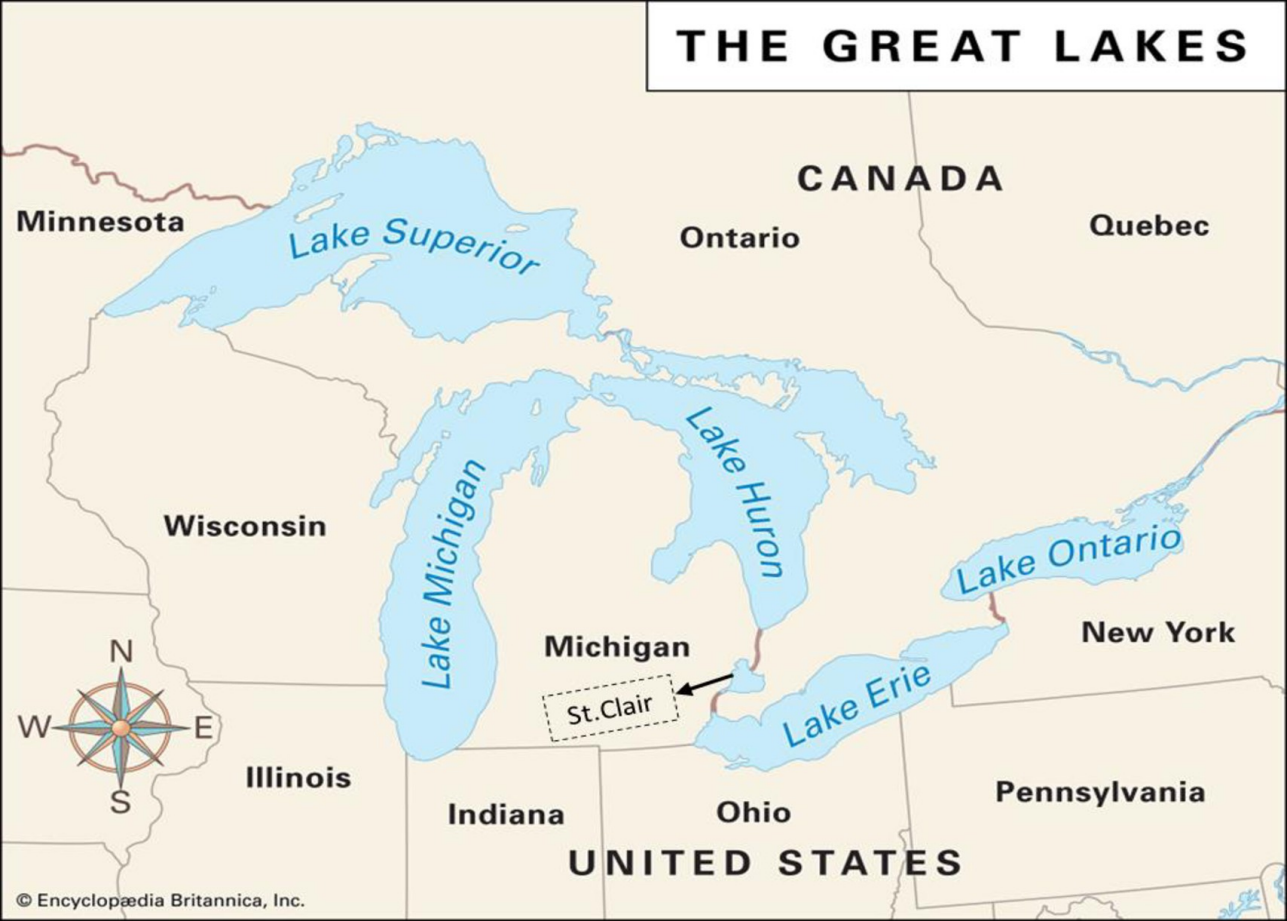
Location of the Great Lakes [1]. 1. Great Lake Map. Available: https://kids.britannica.com/kids/article/Great-Lakes/346130#:—:text = The Great Lakes are five,of fresh water on Earth.

The Great Lakes are a collection of interconnected freshwater lakes with some sea features. They are in the central-eastern part of North America and are linked to the Atlantic Ocean by the Saint Lawrence River. All five lakes—Superior, Michigan, Huron, Erie, and Ontario—are located inside or close to the international boundary between Canada and the United States (see Fig 4 [94]). Lakes Michigan and Huron (Lake Michigan- Huron) are hydrologically considered one body of water since they are connected at the Straits of Mackinac. Furthermore, the Great Lakes Waterway qualifies modern ships to navigate between the lakes. Since approximately one-fifth (21%) of the world’s surface freshwater is contained in the Great Lakes, they are considered the largest group of freshwater lakes on the earth in terms of the total surface area (244,106 km^2^) [95]. The Great Lakes are one of the most significant water resources in the world, and they provide more than fifty million people in eastern North America with water that can be used for various purposes [96]. The five lakes’ cumulative capacity is approximately 6 x 10^15^ gallons, which is enough water to drown North America to an average depth of one meter and indicates how huge the lakes are. It is essential to mention that Lake Superior is the most immense, profound, and upstream of the Lakes [97]. It is important to note that the water level data were acquired and gathered from open access source website [98]. The data covers a long period of time from 1918 to 2021. Notably, the raw data for all lakes are available in B1 Table in S1 File, which can be found in Appendix B.

## 4. Drought calculation

### 4.1 Multivariate standardized water level index (MSWI)

Standardized water level index (SWI) is calculated using the same method as the SPI, but instead of using monthly rain, the water level is used as input [99]. The MSWI is calculated based on several SWI time series for a specific station, and each time scale represents a particular time horizon. The MSWI considers every subset of SWI time scales (i.e., SWI-1, SWI-2, SWI-3, …, to SWI-48 months) for studied lakes. Examining multiple SWI time series in detail can offer valuable insights into drought events. However, comparing and analyzing these time series at different scales can be challenging for scholars and may lead to confusion and inefficiencies in addressing the issues. In addition, it is challenging to precisely define the duration of the timescale, such as long or short-term, and accurately connect it to a specific category of drought impact. For example, short-term drought can be represented on a three-month time scale, which raises the logical question of why a standard drought index cannot define two or four-month scales. Accordingly, the standard drought index may not be derived from a specific criterion to classify some drought time scales (e.g., SWI-4, SWT-5, etc.) as short, medium, or long scale. Given the concerns, some researchers suggested decreasing the number of examined drought aggregation time scales and accompanying series by employing a multivariate approach [100]. Notably, a reduction in SWI series does not mean that the SWI for some time scales are not calculated, but rather that a substantial portion of the variability of the SWI on different time scales is summed up into certain series. Thus, the reduction of these drought time scales is computed by using an effective method called principal component analysis (PCA).

PCA is a widely used prominent approach for analyzing datasets with high-dimensional space (features) [101], providing several benefits such as retaining maximum information while reducing data features, increasing data interpretability, and enabling visualization of multidimensional data. PCA displays the following linear combinations of the *I* original variables:

$${PC}_{k}={E_{k}}^{T}X=\sum_{i=1}^{I}e_{ik}X_{i},i=1,2,3,\ldots ,$$ (35)

In the above Equation, the *PC*_*k*_ is the *k*th principal component while, *E*_*k*_ is the *k*th eigenvector. The original *i*th variable is represented in the above formula as *X*_*i*_ and finally, the *e*_*ik*_ is the *i*th element of the *k*th eigenvector. In PCA, linear combinations are constructed from the original variables. These combinations, known as principal components, are formed in such a way that they are mutually uncorrelated, meaning that they do not have any shared information. The number of principal components that can be generated is determined by the number of original variables in the dataset. The first principal component (*PC*_*1*_) can account for a significant proportion of the variance in the original variables. PCA is particularly useful when there is a high correlation between variables in the dataset. A cross-correlation matrix of the predictors must be constructed to perform PCA, from which the eigenvalues and eigenvectors are calculated. The eigenvector corresponding to the highest eigenvalue represents the weighted coefficients of the first principal component, and this process is repeated for subsequent components.

As *PC*_*1*_ holds the most essential information of the data, the MSWI is derived from this component. However, unlike the standard index (e.g., SWI), which has a mean of zero and a standard deviation of one, *PC*_*1*_ does not meet these requirements due to its algebraic characteristics, which make its values incomparable across different months or locations. Therefore, the time series of *PC*_*1*_ must be standardized using the following Equation based on the mean and standard deviation for various months throughout the year.


$$Z_{1YM}=\frac{{PC}_{1YM}-\bar{{PC}_{1M}}}{{SD}_{1M}}$$ (36)

**Fig 5:**
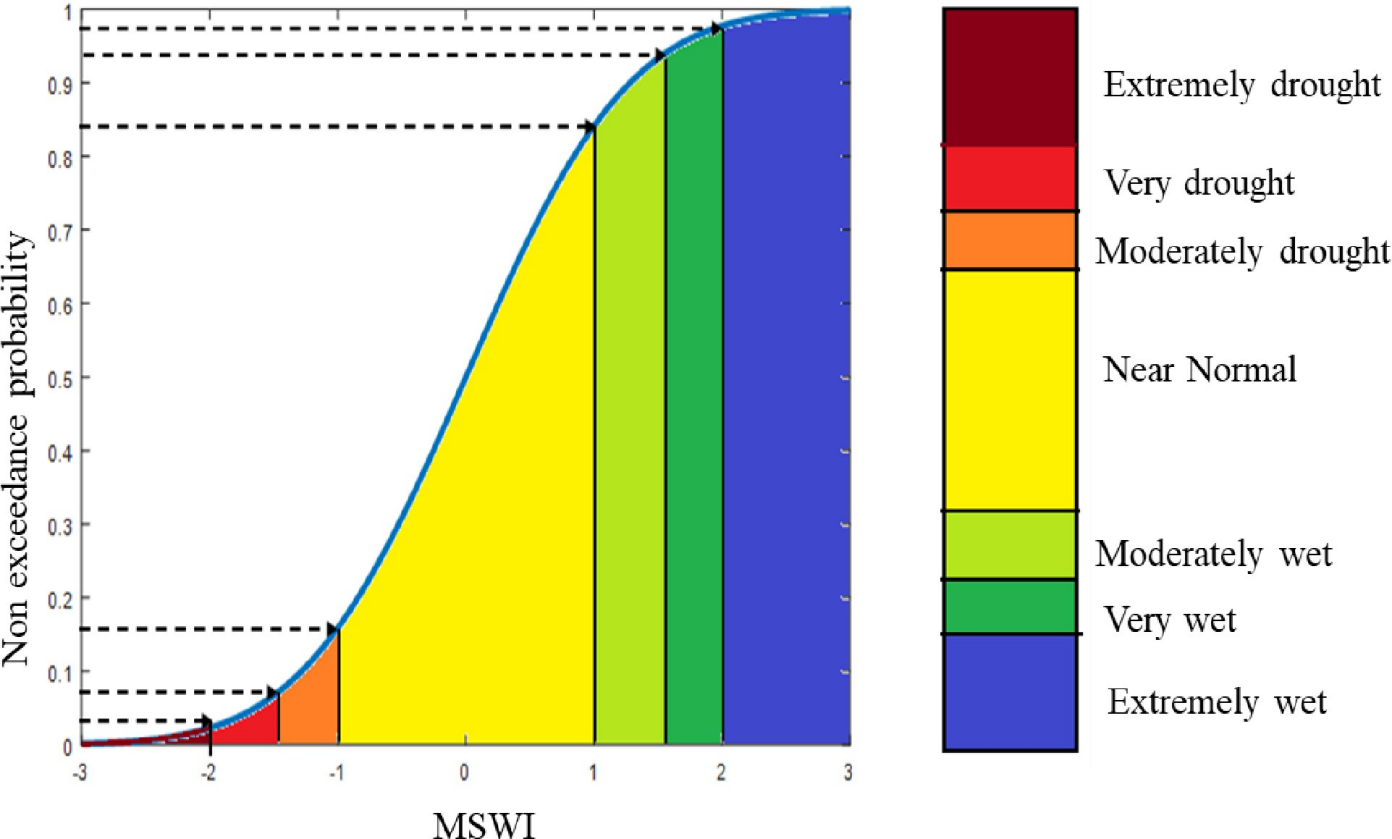
Drought categories based on the MSWI value.

In the Eq 36, the *Z*_1*YM*_ (MSWI) is the *PC*_*1*_ standardized value corresponding to *M*th month and *Y*th year, $\bar{{PC}_{1M}}$, and *SD*_1*M*_ are the average and *PC*_*1*_ component for *M*th month. Notably, the $\bar{{PC}_{1M}}$ value is very small and close to zero; therefore, it can be neglected. The data of MSWI is organized in ascending order, and a graphic of its corresponding empirical probability distribution is shown to identify the categories of drought and wet severity (see Fig 5)[102].

### 4.2. Describing droughts in the Great Lakes

The main focus of this study was on two prominent lakes, specifically Lake Superior and Lake Michigan-Huron, within the forecasting models. These lakes were given significant attention due to their larger size and greater depth, distinguishing them from the other lakes. Besides, they are located upstream, as the water flows from them to the rest of the lakes and then to the sea (see A1 Fig in S1 Appendix). The figures attached in the appendix (A2 Fig-A4 Figs in S1 Appendix) show that in the last fifty years, the three lakes (Lake Erie, Lake Ontario, and Lake St. Clair) did not witness any severe drought events and that the lowest value of the drought indicator is around -0.5. The reason may be related to the dams constructed and the lakes’ applied regulation process. Notably, Lake Superior and Lake Ontario are currently regulated under some plans implemented by the International Joint Commission. Since 1921, Lake Superior’s levels have been regulated, while the water level of Lake Ontario was regulated in 1958. Because there is a significant difference in the elevation, the regulation process of Lake Ontario does not affect the upper lakes’ water level. Also, the regulation of Lake Superior has an important influence on the whole Great Lakes System. Notably, the detailed drought analysis via MSWI for all lakes can be found in B2 Table in S2 File, which is located in Appendix B.

**Fig 6:**
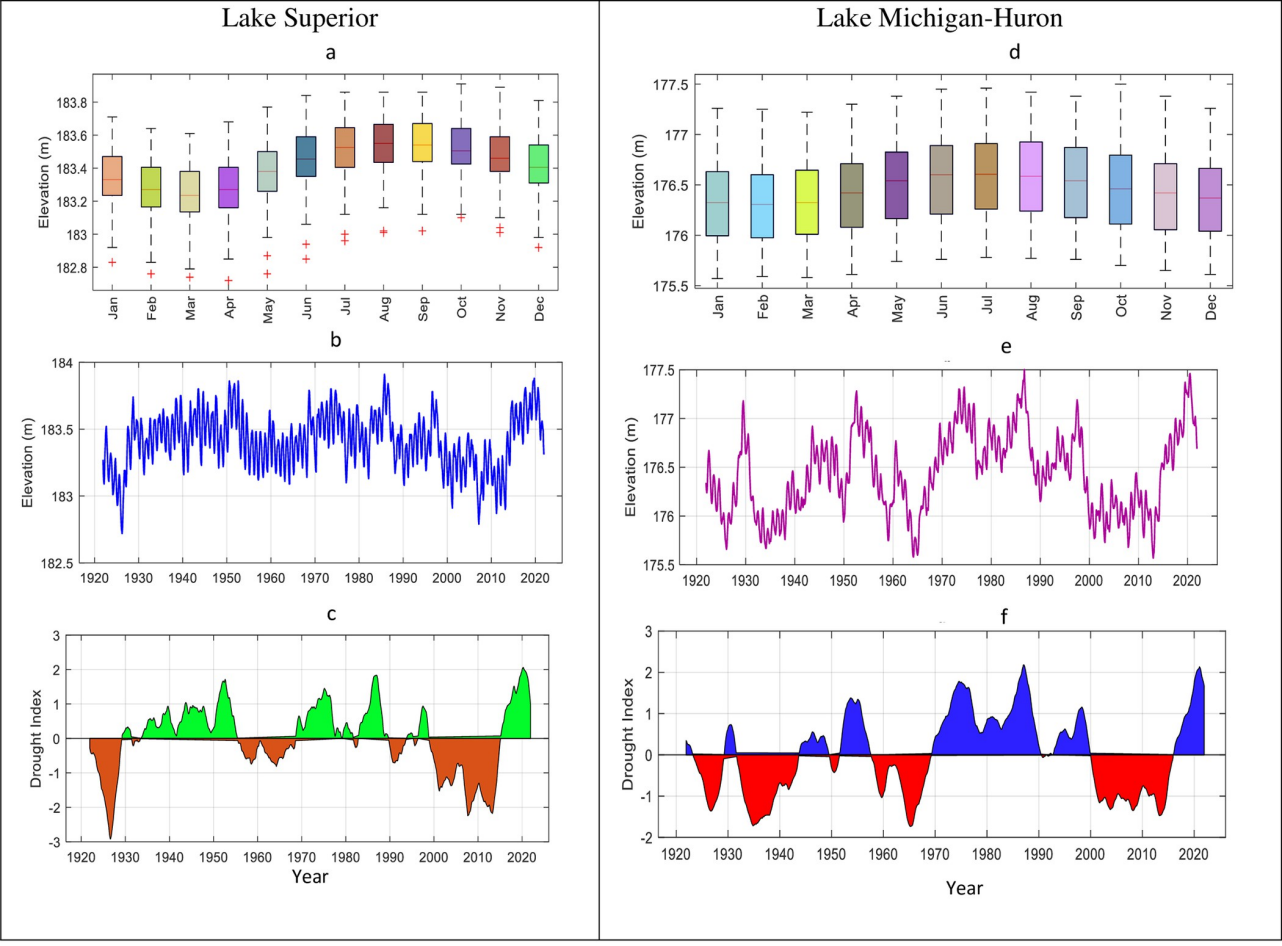
Water level elevation VS drought. a), and d) Boxplot diagrams showing the monthly variation of water level for Lake Superior and Lake Michigan-Huron. b), and c) line graphs showing the yearly water level elevation while c), and f) presenting the MSWI from 1920 to 2020.

According to Fig 6, there is a large variation in the monthly levels of Lake Superior compared to Lake Michigan- Huron. Both lakes have a large area, but the first one is located upstream, and the water flows from it to the other lakes, which significantly varies the monthly levels of stored water. Based on Fig 6C, and 6F, both lakes suffered from severe drought from mid-1921 to 1929 and late 1998 to 2015. Lake Michigan-Huron generally experienced more frequent drought events than Lake Superior; however, the latter faced the severest drought. The analysis also found that Lake Michigan-Huron experienced four severe drought events between 1920 and 2020, while Lake Superior Lake has only faced two drought events during the same period. The droughts of the 1921s and 1929s were primarily caused by reduced precipitation, whereas the drought of the 2000s was mainly attributed to the elevated water temperatures induced by climate change, resulting in enhanced evaporation due to a substantial reduction in winter ice cove [60]. It is essential to mention that the MSWI can explain more than 95% of the variability for the nominated SWI time scales in the observed lakes.

**Fig 7:**
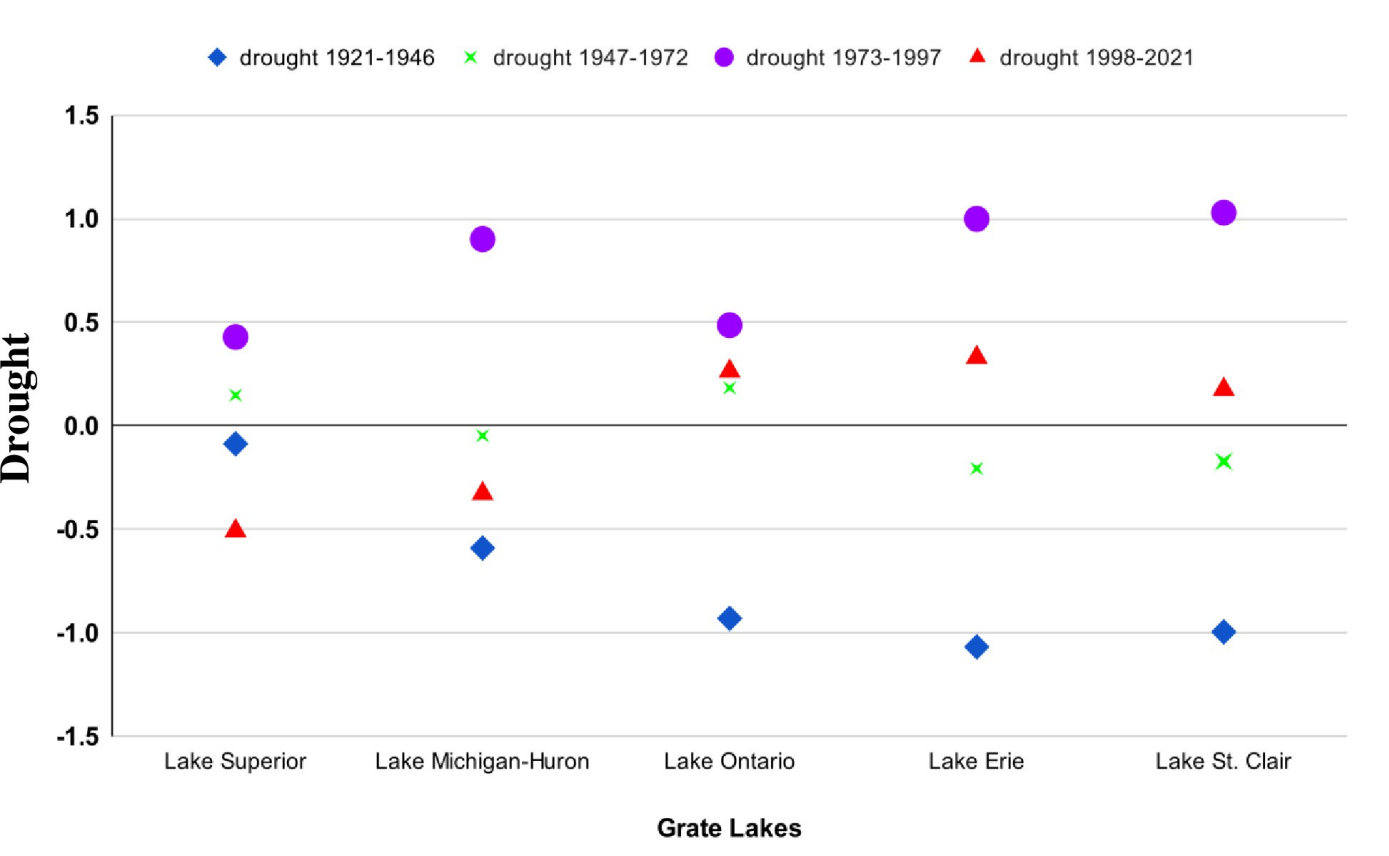
Temporal analysis of drought intensity in the Great Lakes region (1921–2021).

The temporal analysis of drought intensity in the Great Lakes region, as depicted in Fig 7, unveils a dynamic pattern of fluctuating drought conditions from 1921 to 2021. Notably, Lake Superior and Lake Michigan-Huron emerge as critical focal points with notable variations in drought intensity. Conversely, Lake Ontario transitions from drier to wetter conditions, indicating an overall increase in drought values (MSWI) and a positive drought trend. Similarly, Lake Erie experiences a substantial transition towards positive drought conditions. Lake St. Clair also exhibits patterns consistent with the other lakes. This comprehensive analysis demonstrates the diverse drought intensities observed across the Great Lakes over the analyzed period, particularly emphasizing the significant decrease in drought observed in Lake Superior and Lake Michigan-Huron from 1998 to 2021. This pronounced finding highlights a significant shift in drought conditions for these lakes, underscoring the region’s evolving nature of drought dynamics.

## 5. Results and discussion

The results section is divided into two main scenarios. In the first scenario, the RELM-BWO model is validated against standalone models, while the second scenario compares the performance of the RELM-BWO model with other hybrid models. Since severe drought periods were observed in only Lake Superior and Lake Michigan-Huron over the past fifteen years, the models were compared, and the best performing one was selected to forecast drought over the remaining three lakes.

### 5.1 First Scenario: A comparison of RELM-BWO with classical models

#### 5.1.1 Modelling results

This section discusses the evaluated perfo7rmance of four applied models (RF, SVR, RLM, and RELM-BWO) in forecasting MSWI time series for different lead times using various statistical measures and comparable graphs for Lake Superior and Lake Michigan-Huron. The performance measures over the testing phase for both lakes are provided in Tables 2 and 3. The provided assessment shows that the best forecasting result for Lake Superior was obtained for RELM-BWO (*R*^*2*^ = 0.9687, *RMSE* = 0.0202, and *MAE* = 0.0156) compared to the other drought forecasting models. Similarly, the adopted model performs better in forecasting one-step month ahead for Lake Michigan-Huron, attaining higher accuracy (*R*^*2*^ = 0.9825, *RMSE* = 0.0117, and *MAE* = 0.0093). Generally, the quantitative assessment showed that the performance of the forecasting models in Lake Superior is slightly lower than in Lake Michigan-Huron. Furthermore, the second-best model for forecasting drought is ELM, followed by SVR and the RF shows the poorest performance.

**Table 2:** Performance indicators of machine learning-based models for Lake superior.

Model	Performance metrics	Period lead time					
		1	2	3	4	5	6
**SVR**	MAE	0.0497	0.0706	0.0867	0.0867	0.1596	0.1956
	RMSE	0.0771	0.0887	0.1089	0.1289	0.1974	0.2380
	MAPE %	5.53	9.34	12.11	12.14	26.62	35.74
	R^2^	0.9320	0.9299	0.9256	0.9256	0.9002	0.8854
**RF**	MAE	0.2126	0.2338	0.2768	0.3305	0.3148	0.3191
	RMSE	0.2746	0.3086	0.3521	0.3969	0.3822	0.3838
	MAPE %	32.14	37.61	41.83	49.44	43.05	56.20
	R^2^	0.9280	0.9210	0.9000	0.8983	0.8825	0.8720
**ELM**	MAE	0.0206	0.0462	0.0767	0.0944	0.1443	0.1873
	RMSE	0.0265	0.0601	0.0976	0.1229	0.1774	0.2329
	MAPE %	2.46	6.15	10.45	14.31	28.98	36.72
	R^2^	0.9561	0.9499	0.9561	0.9375	0.9252	0.8942
**RELM-BWO**	MAE	0.0156	0.0371	0.0587	0.0886	0.1191	0.1524
	RMSE	0.0202	0.0489	0.0773	0.1149	0.1523	0.1919
	MAPE %	2.00	4.93	8.44	13.61	20.31	30.62
	R^2^	0.9687	0.9601	0.9572	0.9457	0.9342	0.9179

**Table 3:** Performance indicators of machine learning-based models for Lake Michigan-Huron.

Model	Performance metrics	Period lead time					
		1	2	3	4	5	6
**SVR**	MAE	0.0222	0.0361	0.0438	0.0610	0.0806	0.1084
	RMSE	0.0275	0.0434	0.0533	0.0751	0.0994	0.1338
	MAPE %	6.28	7.41	7.77	10.41	13.31	17.11
	R^2^	0.9709	0.9669	0.9652	0.9752	0.9529	0.9352
**RF**	MAE	0.0798	0.1159	0.1303	0.0939	0.1093	0.1309
	RMSE	0.1151	0.1748	0.1846	0.1162	0.1401	0.1688
	MAPE%	15.19	24.11	28.01	18.89	20.01	23.06
	R^2^	0.9608	0.9379	0.9300	0.9568	0.9465	0.9278
**ELM**	MAE	0.0138	0.0259	0.0396	0.0528	0.0696	0.0913
	RMSE	0.0175	0.0321	0.0488	0.0652	0.0862	0.1129
	MAPE %	3.17	6.31	7.23	8.43	14.37	16.19
	R^2^	0.9737	0.9684	0.9640	0.9597	0.9569	0.9503
**RELM-BWO**	MAE	0.0093	0.0209	0.0339	0.0490	0.0670	0.0812
	RMSE	0.0117	0.0258	0.0416	0.0611	0.0822	0.1005
	MAPE %	2.82	5.76	7.69	8.16	13.44	15.45
	R^2^	0.9825	0.9791	0.9772	0.9736	0.9681	0.9639

The evaluated results showed that the forecasting accuracy of the models reduces when the lead time increases. The forecasting results generally deteriorate with the increase in forecasting multistep month ahead drought due to accumulated errors. For example, for Lake Superior, the RF model is considered among the models the most influenced by the change of time interval where the *MARE%* varies from 32.14 to 56.20% followed by SVR (5.53 to 35.74%), ELM (2.46 to 36.72%), and RELM-BWO (2 to 30.62%). Regarding Lake Michigan-Huron, all models, except the RF, suffered from a gradual decrease in performance with the increase in lead time. It can be observed that these models have similar characteristics in forecasting the drought for both lakes, producing the peak errors in the forecasting drought for a six-month ahead lead time. The RF has an abnormal performance for forecasting drought at Lake Michigan-Huron as it generates the maximum error at the fourth-step lead time. However, the RF has the lowest forecasting accuracy for all time intervals compared to other models for both lakes.

**Fig 8:**
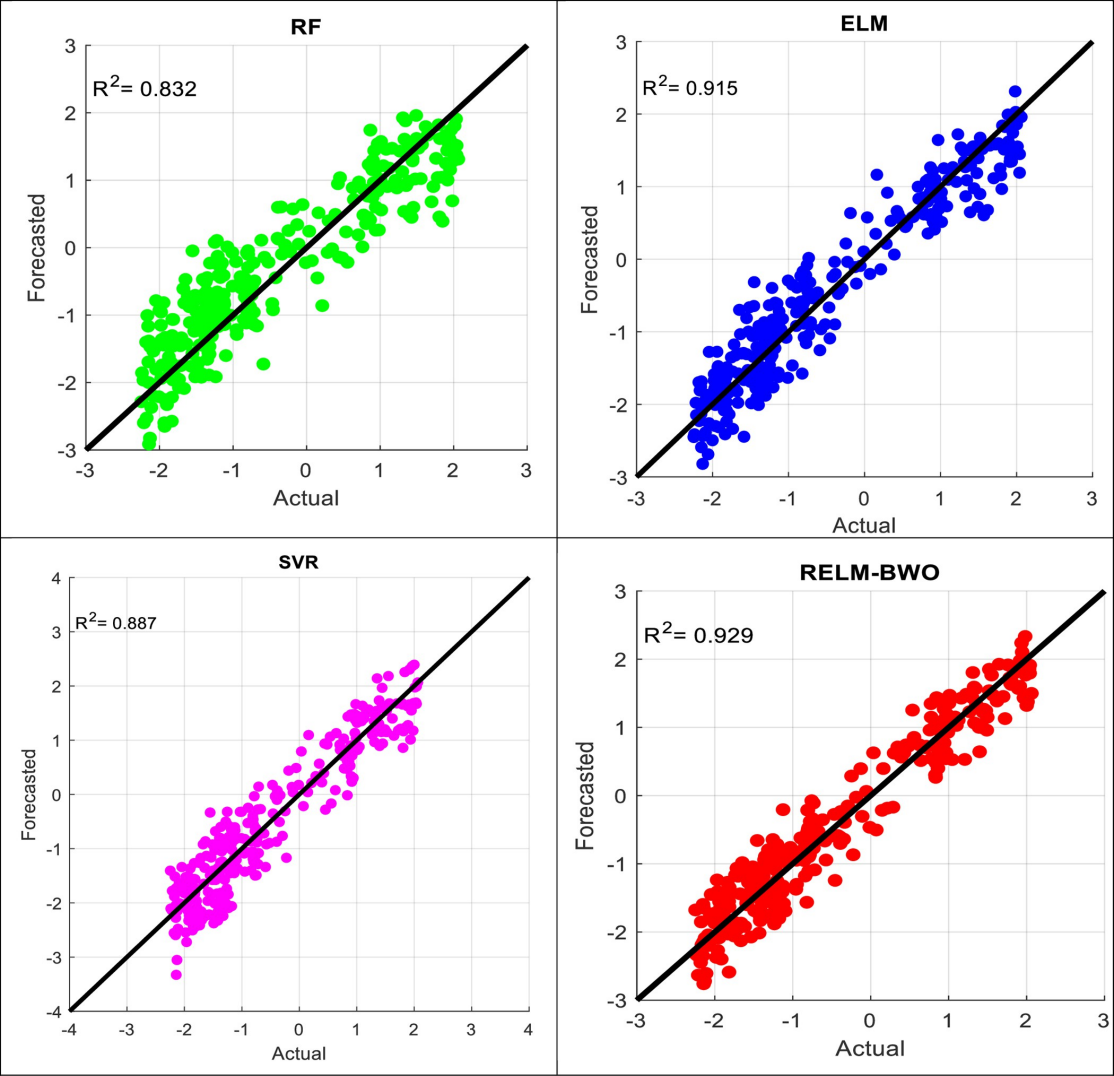
A comparison of observed MSWI and forecast values throughout a six-lead period time for Lake Superior.

**Fig 9:**
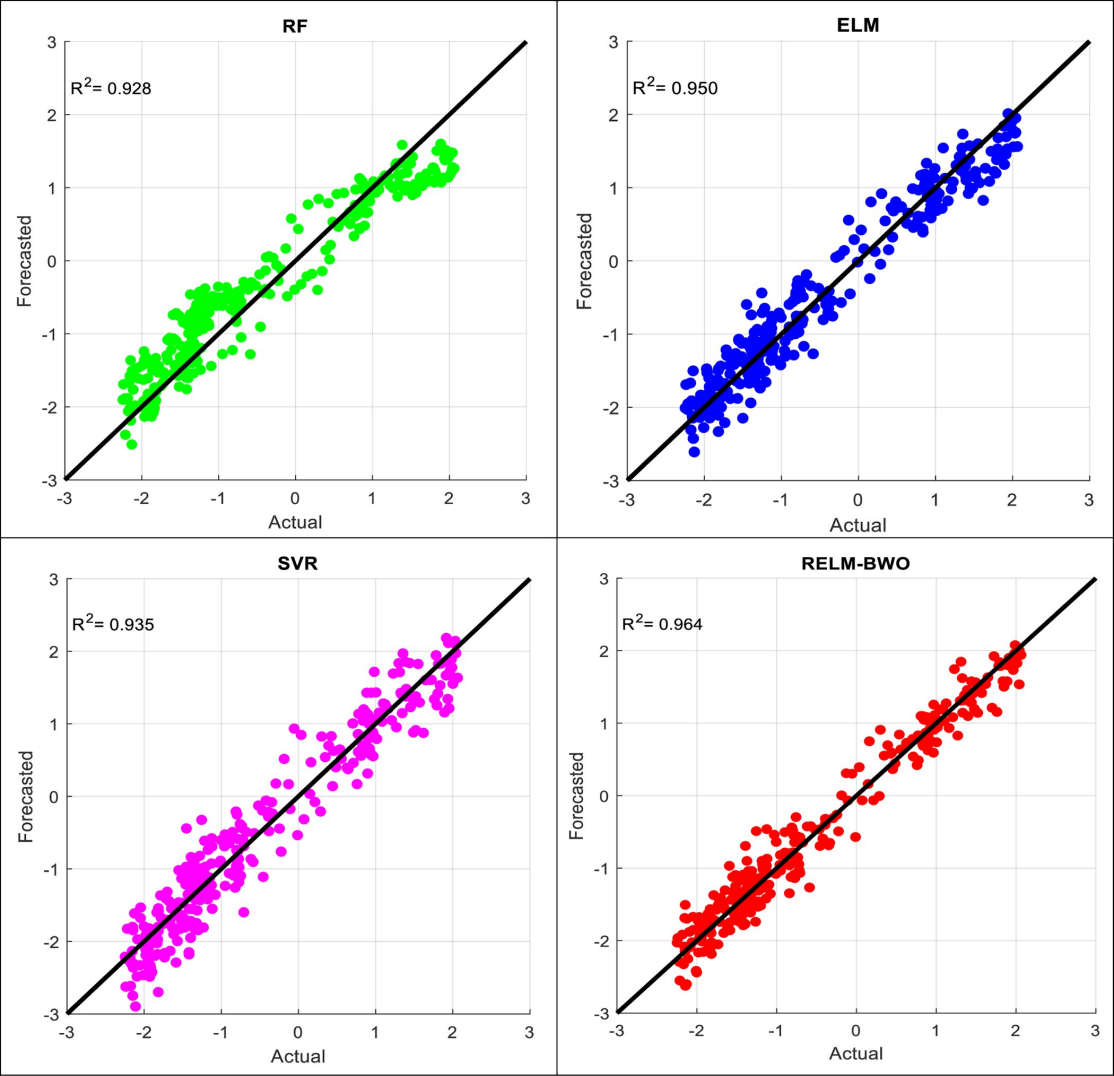
A comparison of observed MSWI and forecast values throughout a six-lead period time for Lake Michigan-Huron.

The quantitative analysis, in general, shows that the most difficult case for the adopted models is forecasting drought at a six-period lead time. In other words, forecasting drought for the next six months is a major challenge for the proposed models because of the loss of important information as well as the sharp changes that occur in drought patterns through such a period, which was revealed by the analyzes previously reported, so it is necessary to test the efficacy of the models in this case. Accordingly, scatter plot diagrams, as shown in Figs 8 and 9 for both lakes, are created. Such a diagram allows us to visualize each data record and provides much information on how the forecasting values deviate from their corresponding actual values. The figures show that the suggested model offers fewer scatter points than comparable models. It is significant to conclude that the forecasting accuracy of Lake Michigan-Huron is higher than Lake Superior. The reasons may relate to the training conditions of the forecasting models. In this study, the models were calibrated using 75% of the drought time series data, while the remaining 25% of the hydrological drought data was reserved for testing purposes. The training data for Lake Michigan-Huron includes many positive and negative drought values, and the characteristics of the training data are more similar to the test data compared to Lake Superior (see Fig 6C and 6F). In more detail, the training data for Lake Superior contained a small percentage (5.88%) of the severe drought data (MSWI ≤ -1), while that value approximately tripled (16.32%) when it came to Lake Michigan-Huron. To sum it up, the presence of multiple periods of severe drought conditions enhances the chance of obtaining accurate forecasts. Conversely, when such observations are few in the training data, it is normal for a forecasting model to observe a decrease in accuracy, especially when asked to simulate severe drought patterns.

**Fig 10:**
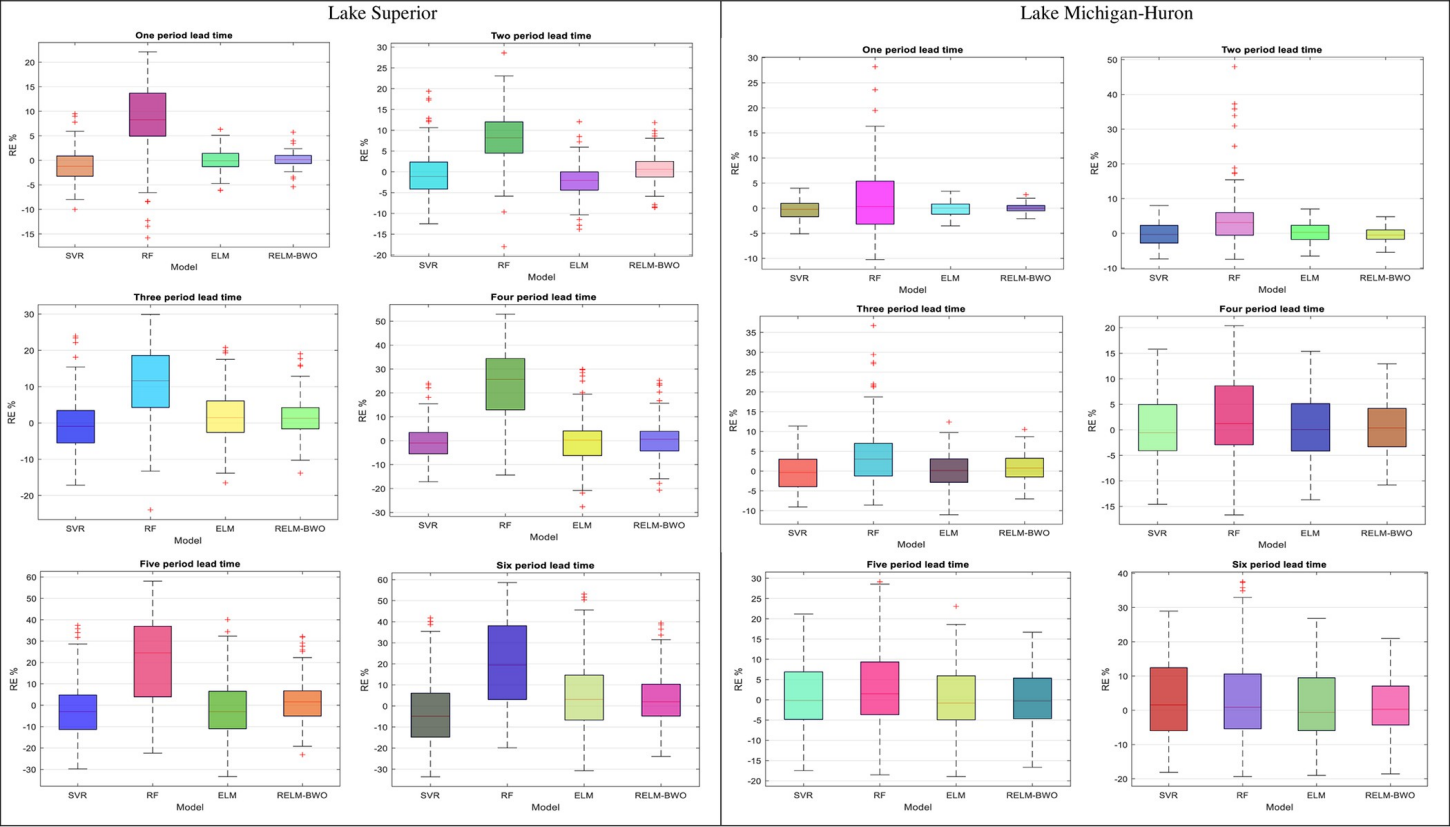
Relative error showing the forecasting accuracy of SVR, RF, ELM and RELM-BWO models over different lead times for Lake Superior and Lake Michigan-Huron.

Assessing the efficacy of the proposed models is crucial, particularly during the severe drought period (MSWI ≤ -1). The RE% values for each model are calculated (as illustrated in Fig 10) across various lead-time intervals to determine the practical benefits of the RELM-BWO model concerning its forecasting precision and performance compared to other models. The proposed RELM-BWO model generally outperforms other comparable methods, with the median relative error (depicted as a horizontal line within the boxplot) being lower.

#### 5.1.2 Analyzing forecasts of turning points

The performance metrics listed in Tables 2 and 3 evaluate a model’s overall performance by considering all predicted points. However, when forecasting drought, metrics such as *RMSE*, *R*^*2*^, and others may not be ineffective indicators due to high auto-correlation. An overly smoothed time series is unsuitable for testing a prediction model’s effectiveness or robustness due to high auto-correlation. This can lead to *R*^*2*^ values exceeding 0.9 for all models, which may not be significant for drought forecasting unless the model can accurately forecast turning points. Hence, assessing the error in turning points of the drought time series data is critical to gauge the effectiveness of models used in forecasting drought.

**Fig 11:**
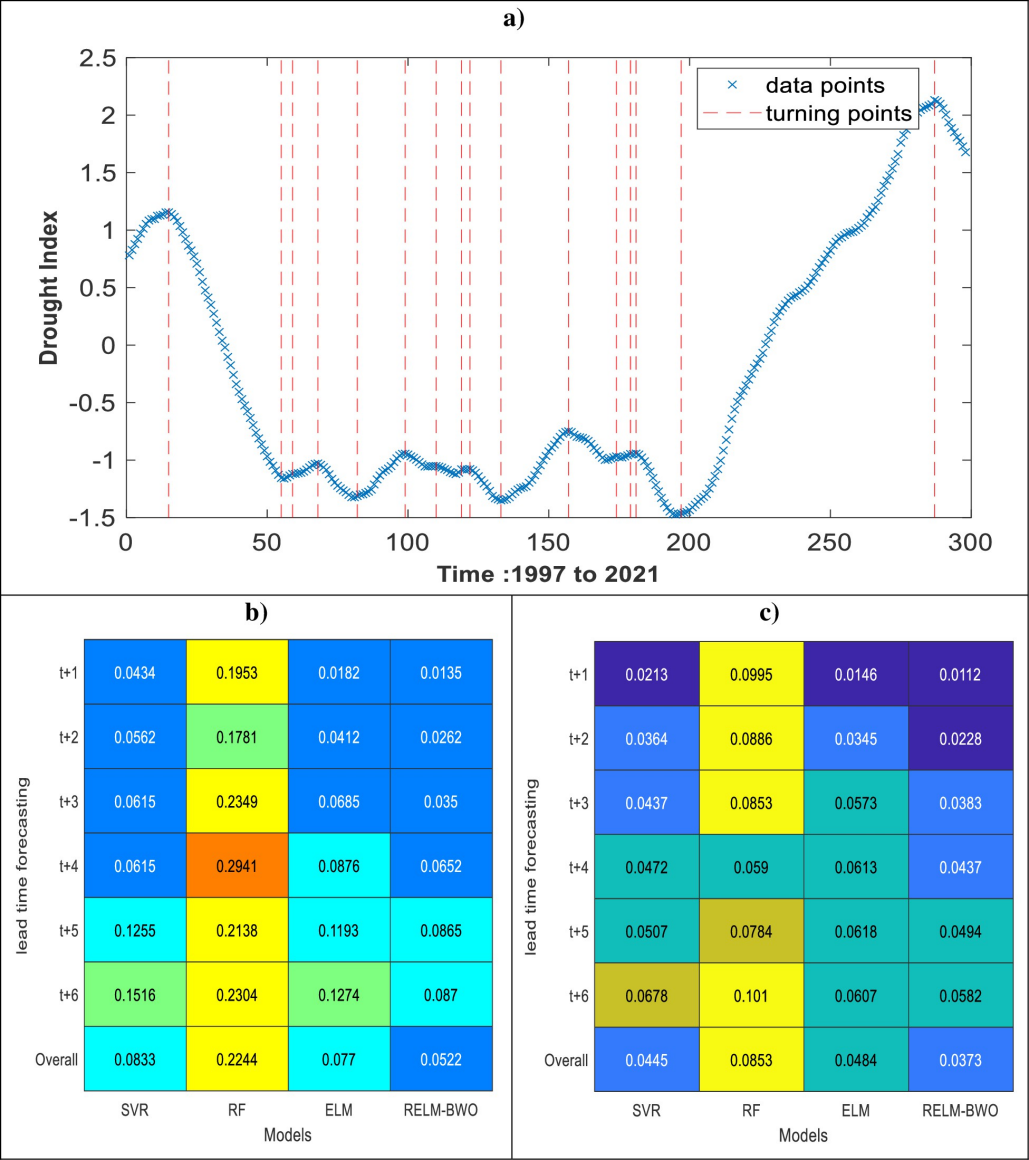
Analysis of the errors in the turning and critical points at different lead time forecasting. a) an example of detecting the turning points, b) results of AAETP for Lake Superior, and c) results of AAETP for Lake Michigan-Huron.

The primary objective of this study’s method was to evaluate the model’s ability to accurately simulate turning points in the drought data. An illustration of the process for identifying critical and turning points in time series data can be found in Fig 11A. Once the these points were detected, the corresponding observations from multiple models were collected. The final step involved calculating the average absolute error of the turning and critical points (*AAETP*) for each model separately. It is significant to mention that the suggested process was conducted for both lakes to assess the model’s predictability in forecasting hydrological drought up to six months in advance.

$$AAETP=average\;(|E_{i}|)$$ (37)

where *E*_*i*_ is a vector is computed for each model by subtracting the actual values of the critical and turning points from the values forecasted by the models.

In Fig 11B and 11C, it was demonstrated that the RELM-BWO model outperformed other models in forecasting turning points and yielded fewer *AAETP* values. The RELM-BWO model’s effectiveness is measured by its ability to decrease the *AAETP* in drought forecast for both lakes at different lead times. The study’s findings show that the RELM-BWO model outperforms traditional models like SVR, RF, and ELM for Lake Superior, resulting in a 37.33%, 76.74%, and 32.21% improvement in forecasting accuracy. However, the accuracy of drought forecasting for Lake Michigan-Huron decreases slightly, but the RELM-BWO model still outperforms SVR, RF, and ELM by reducing forecasting errors by 16.18%, 56.27%, and 22.93%, respectively.

### 5.2 Second Scenario: Validation of the hybrid models

This section presents a comparison of four hybrid models, namely RELM-BWO, SVR-BWO, RF-BWO, and ELM-BWO, to forecast drought conditions over one to six months in advance for the two largest lakes. The performance of each model in forecasting drought, considering various lead times, is demonstrated in Table 4. The statistical analysis revealed that the RELM-BWO model outperforms other models in terms of drought forecasting, exhibiting the lowest values for *MAE* (ranging from 0.0156 to 0.1524), *RMSE* (ranging from 0.020 to 0.191), *MAPE* (ranging from 2 to 30.62%), and highest *R*^*2*^ (ranging from 0.9179 to 0.9687) for Lake Superior. Similarly, for Lake Michigan-Huron, RELM-BWO demonstrates the lowest values for *MAE* (ranging from 0.0093 to 0.0812), *RMSE* (ranging from 0.0117 to 0.1005), *MAPE* (ranging from 2.82 to 15.45%), and highest *R*^*2*^ (ranging from 0.9639 to 9825). Overall, the RELM-BWO model is considered the best-performing model, followed by ELM-BWO, SVR-BWO and RF-BWO, respectively.

**Table 4:** Performance indicators of the hybrid machine learning-based models.

Model	Lake Superior						Lake Michigan-Huron				
	Lead Time	MAE	RMSE	MAPE%	R^2^	AAETP	MAE	RMSE	MAPE%	R^2^	AAETP
**ELM-BWO**	t+1	0.0194	0.0238	2.34	0.9602	0.0165	0.0119	0.0156	2.94	0.9801	0.0140
	t+2	0.0381	0.0576	5.62	0.9579	0.0335	0.0233	0.0293	6.07	0.9745	0.0275
	t+3	0.0701	0.0942	9.06	0.9434	0.0450	0.0370	0.0463	7.12	0.9730	0.0427
	t+4	0.0865	0.1218	12.63	0.9291	0.0793	0.0508	0.0647	8.38	0.9703	0.0441
	t+5	0.1323	0.1659	25.90	0.9239	0.0949	0.0684	0.0831	14.28	0.9666	0.0581
	t+6	0.1675	0.2073	35.35	0.9101	0.1095	0.0891	0.1097	15.97	0.9602	0.0655
	Overall	0.0857	0.1118	15.15	0.9374	0.0631	0.0468	0.0581	9.13	0.9708	0.0420
**SVR-BWO**	t+1	0.0432	0.0686	4.04	0.9524	0.0384	0.0175	0.0216	3.85	0.9794	0.0203
	t+2	0.0557	0.0736	5.96	0.9502	0.0496	0.0268	0.0330	5.59	0.9744	0.0309
	t+3	0.0709	0.0942	12.23	0.9473	0.0572	0.0403	0.0498	7.73	0.9725	0.0391
	t+4	0.0684	0.1188	11.15	0.9446	0.086	0.0592	0.0725	10.01	0.9694	0.0411
	t+5	0.1505	0.1848	24.83	0.9323	0.0985	0.0768	0.0943	13.32	0.9649	0.0489
	t+6	0.1848	0.2136	32.45	0.9091	0.1412	0.1020	0.1252	17.48	0.9573	0.0609
	Overall	0.0956	0.1256	15.11	0.9393	0.0785	0.0538	0.0661	9.66	0.9697	0.0402
**RF-BWO**	t+1	0.0626	0.0844	10.84	0.9514	0.0545	0.0280	0.0352	7.63	0.9754	0.0280
	t+2	0.1026	0.1282	17.65	0.9484	0.0824	0.0454	0.0564	11.78	0.9739	0.0433
	t+3	0.1329	0.1700	22.48	0.9442	0.0804	0.0661	0.0818	13.74	0.9711	0.0601
	t+4	0.1907	0.2324	33.72	0.9404	0.1387	0.0860	0.1089	14.41	0.9665	0.0698
	t+5	0.2230	0.2690	37.27	0.9232	0.1564	0.1062	0.1376	16.98	0.9599	0.0792
	t+6	0.2713	0.3287	46.08	0.8907	0.1785	0.1249	0.1604	24.65	0.9487	0.0883
	Overall	0.1639	0.2021	28.01	0.9331	0.1152	0.0761	0.0967	14.87	0.9659	0.0615
**RELM-BWO**	t+1	0.0156	0.0202	2.00	0.9687	0.0135	0.0093	0.0117	2.82	0.9825	0.0112
	t+2	0.0371	0.0489	4.93	0.9601	0.0262	0.0209	0.0258	5.76	0.9791	0.0228
	t+3	0.0587	0.0773	8.44	0.9572	0.0350	0.0339	0.0416	7.69	0.9772	0.0383
	t+4	0.0886	0.1149	13.61	0.9457	0.0652	0.0490	0.0611	8.16	0.9736	0.0437
	t+5	0.1191	0.1523	20.31	0.9342	0.0865	0.0670	0.0822	13.44	0.9681	0.0494
	t+6	0.1524	0.1910	30.62	0.9179	0.0870	0.0812	0.1005	15.45	0.9639	0.0582
	Overall	0.0786	0.1008	13.32	0.9473	0.0522	0.0436	0.0538	8.89	0.9741	0.0373

The RELM-BWO model’s accuracy has been tested against other hybrid models by measuring its ability to reduce the *AAETP* indicator for various lead times in the selected lakes. The overall results, which are presented in Table 4, demonstrate that the RELM-BWO model outperforms the ELM-BWO, SVR-BWO, and RF-BWO models, respectively, for Lake Superior. This results in a significant improvement in forecasting accuracy of 17.27%, 29.65%, and 54.69%. Similarly, for Lake Michigan-Huron, the RELM-BWO model was able to significantly decrease the *AAETP* indicator percentage values, resulting in an 11.19%, 7.21%, and 39.35% improvement compared to the ELM-BWO, SVR-BWO, and RF-BWO models, respectively.

**Fig 12:**
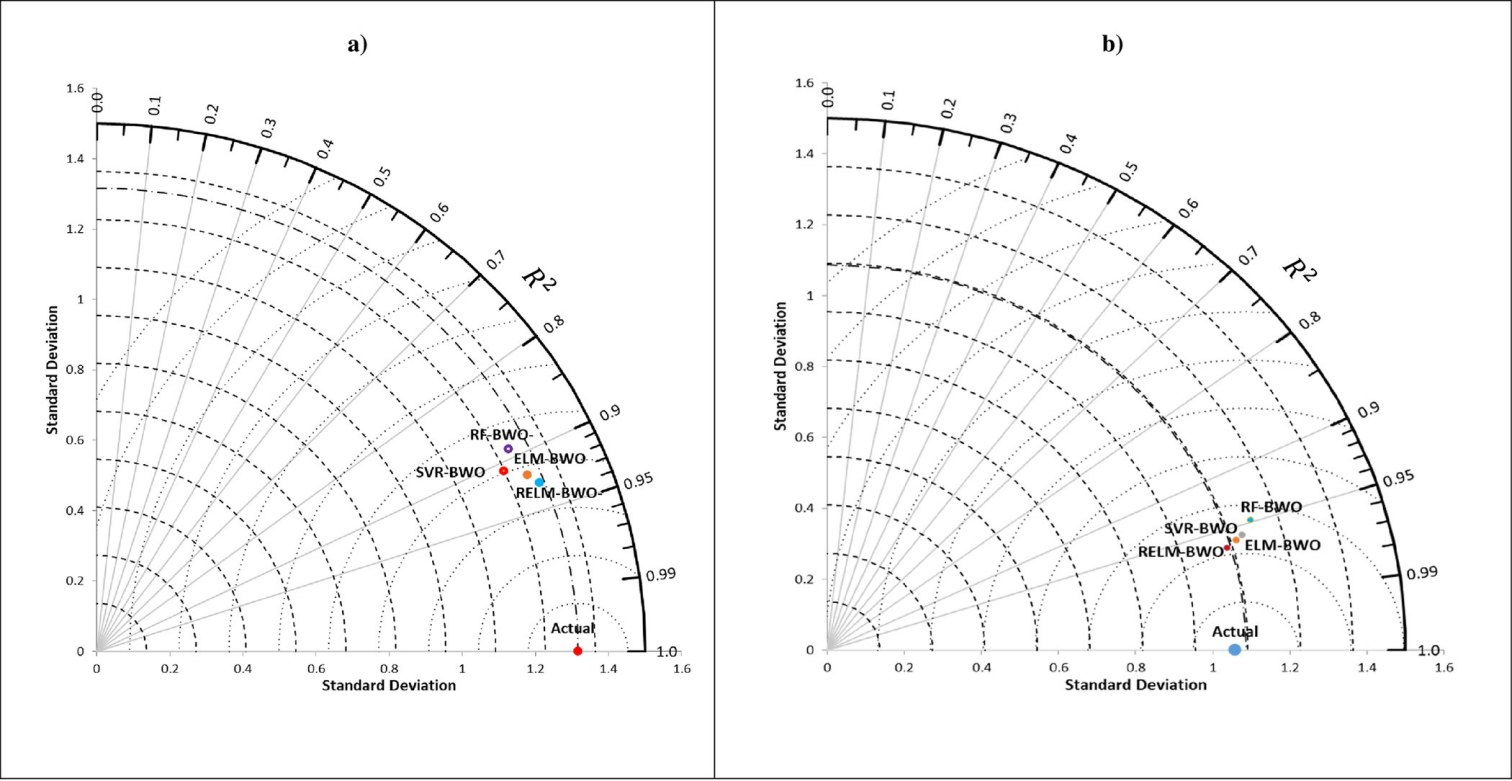
Comparing hybrid models in forecasting MSWI using Taylor diagrams. a) Lake Superior and b) Lake Michigan-Huron.

**Fig 13:**
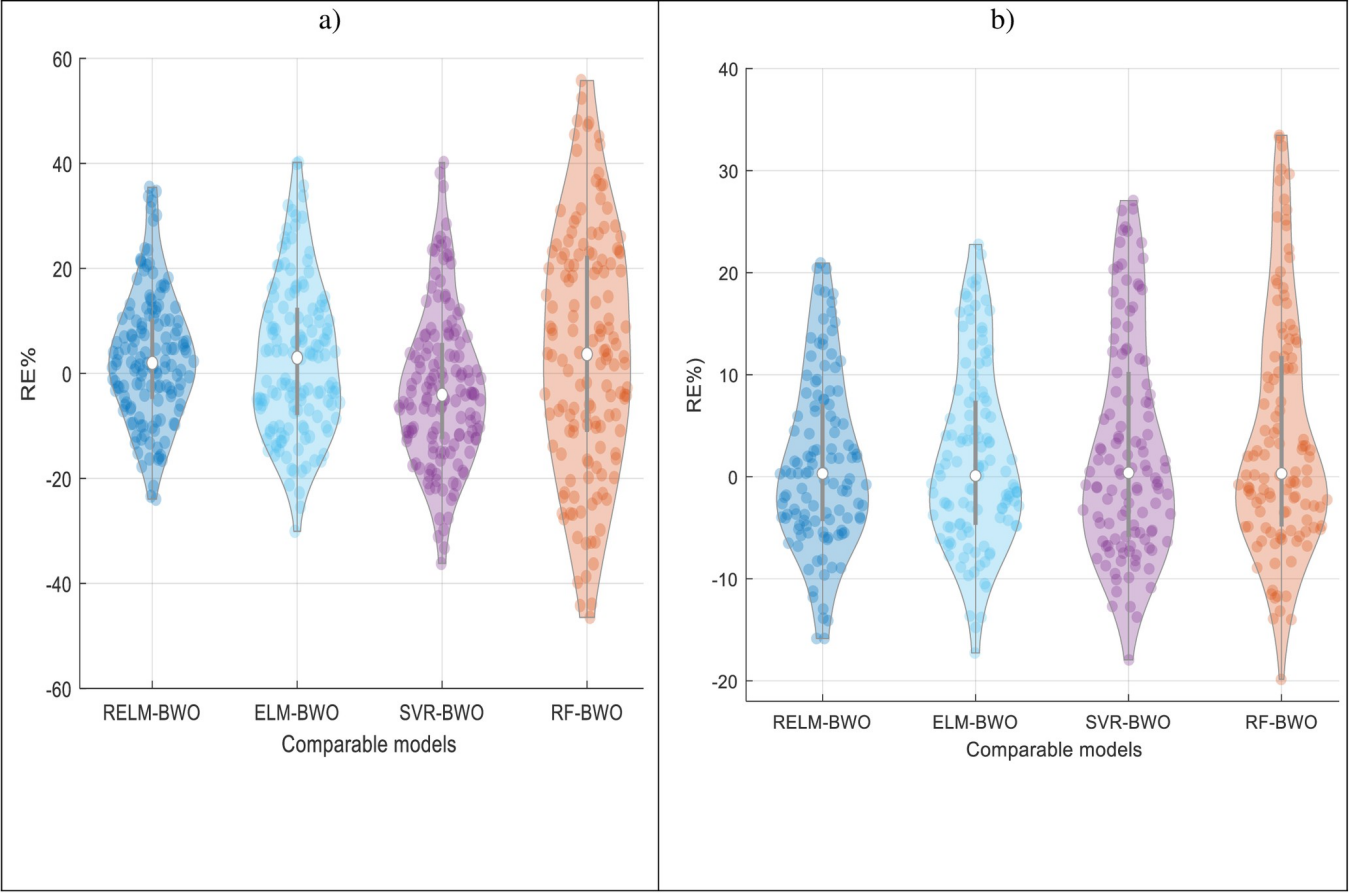
Comparing hybrid models in forecasting MSWI using Violin plots. a) Lake Superior and b) Lake Michigan-Huron.

The accuracy of drought forecasting six months in advance, as exemplified by the hybrid models, has been visually analyzed using Taylor plots (refer to Fig 12) and Violin plots (refer to Fig 13). Taylor plots reveal that the RELM-BWO model demonstrates a closer standard deviation to the calculated drought than other models, with the highest *R*^*2*^ value and the lowest *RMSE* for both lakes. Evaluating the effectiveness of these models, especially during critical drought periods (MSWI ≤ -1), is essential. The *RE%* values of each model have been calculated (as depicted in Fig 13) to ascertain the practical advantages of the RELM-BWO model in terms of its forecasting accuracy and performance compared to other models. The proposed RELM-BWO model generally outperforms hybrid models, with a lower median relative error (indicated by the white hollow point near the Figure’s centre) and a smaller interquartile range (a grey line at the Figure’s core).

### 5.3 Validation of RELM-BWO: A comparative analysis against previous models in literature

A more thorough analysis is needed to confirm the superiority of the proposed RELM-BWO model in forecasting drought one month ahead. This requires verifying the accuracy of the RELM-BWO model by comparing its performance with effective models developed in previous studies. One such study employed sophisticated models that combined wavelet transform (WA) with ANFIS, resulting in the WANFIS model [103]. The study’s conclusion indicates that the WANFIS model performs satisfactorily in forecasting mid-term drought, achieving the highest accuracy with an *R*^*2*^ value of 0.9133. Another study examined a model created by merging WA and ANN, which achieved an impressive *R*^*2*^ of 0.938 [104]. Additionally, an innovative approach was developed by combining LSTM and ARIMA models to enhance the accuracy of hydrological drought forecasts [105]. The integration of these models resulted in a hybrid model that achieved an impressive *R*^*2*^ score exceeding 0.91. Furthermore, a separate study utilized an advanced forecasting model that combined Online Sequential ELM with two decomposing filters, namely "Kalman filter regression and coupled with the boundary corrected maximal overlap discrete wavelet transform" [1], to forecast drought in Iran and the model demonstrated a high accuracy with an *R*^*2*^ of 0.93. Furthermore, a hybrid model incorporating ANN, SARIMA, and a genetic algorithm was employed to forecast hydrological drought, yielding a good *R*^*2*^ of 0.945 [106].

Other researchers have introduced advanced models using Fuzzy-SVR, Boosted-SVR, and classical SVR for drought forecasting, with the Fuzzy-SVR model performing the best with an *R*^*2*^ of 0.903 [107]. Additionally, other researchers have examined individual models for drought forecasting, such as ANN, achieving good results (*R*^*2*^ > 0.9) [62]. Lastly, a sophisticated combined model was developed by integrating WA, ARIMA, and ANN, resulting in a higher *R*^*2*^ of 0.872 [108]. In summary, the models assessed demonstrated satisfactory accuracy, with *R*^*2*^ values ranging from 0.872 to 0.945. However, these models’ forecasting accuracy remains lower than that of the RELM-SO model, which boasts a superior forecasting accuracy (*R*^*2*^ > 0.968).

### 5.4 Results of uncertainty analysis (UA)

According to the previous discussions and the results presented, it can be confirmed that the RELM-BWO has the best performance among the employed models, obtaining the most accurate forecasts. Even though this model is superior, the forecasting accuracy gradually decreases when the lead time increases. Hence, it is critical to determine the maximum lead time for reliable drought forecasting that the proposed model can provide. Therefore, it is necessary to conduct the uncertainty analysis to ensure the reliability and validity of the results obtained.

**Fig 14:**
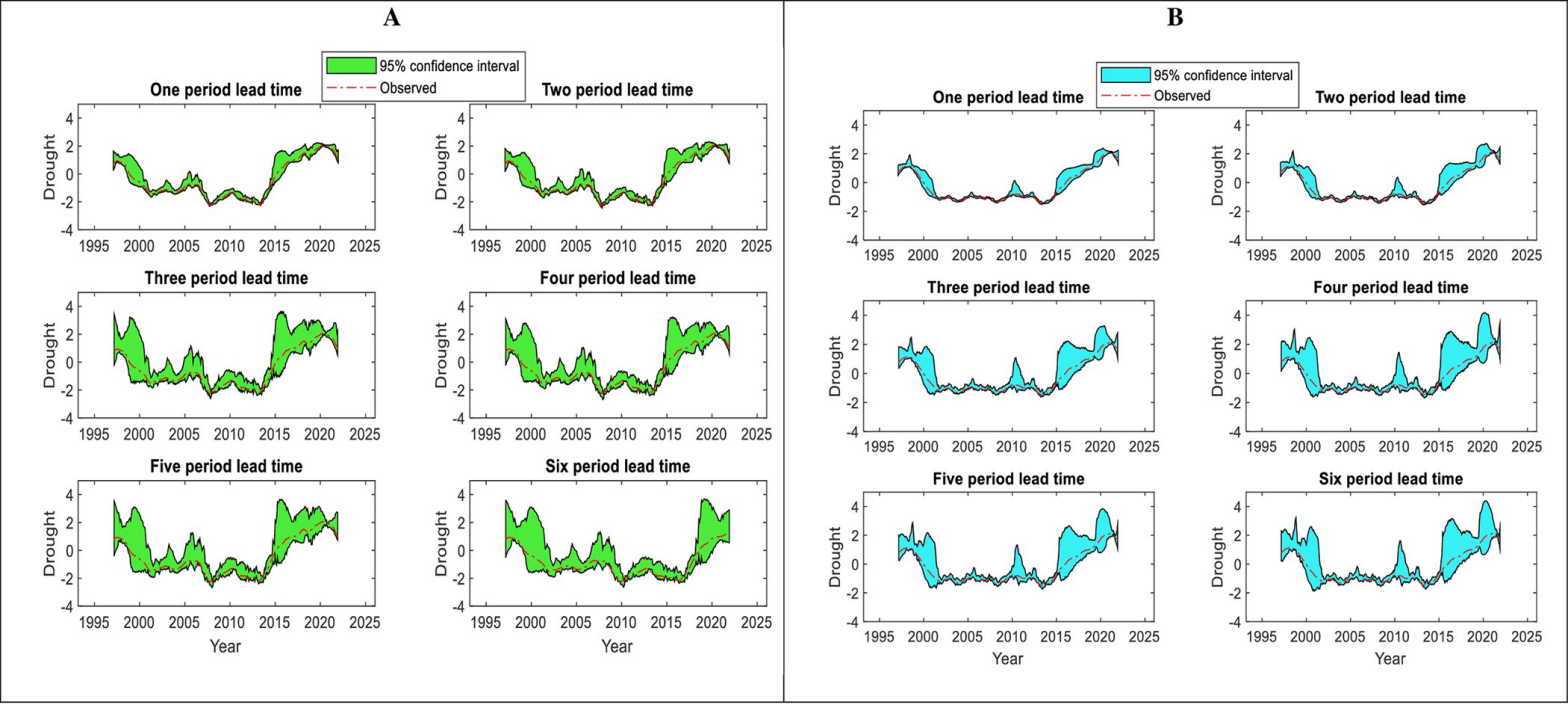
Uncertainty analysis showing the confidence intervals of drought index for A) Lake Superior, and B) Lake Michigan-Huron.

**Fig 15:**
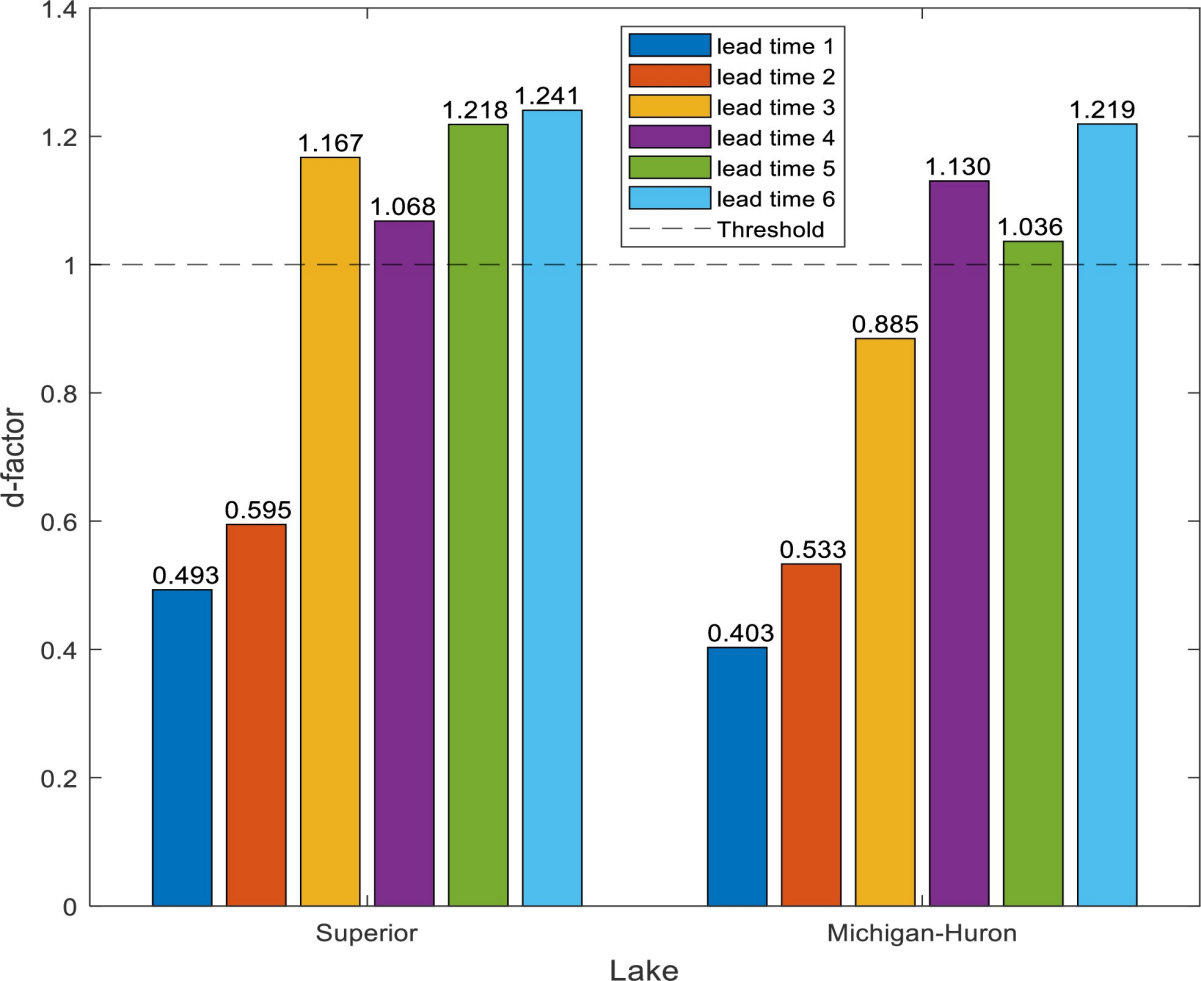
Values of d-factor showing the reliable accuracy of forecasted drought event based on lead time intervals for Lake Superior and Lake Michigan-Huron.

UA results provide much information on how the variations of forecasting results take place due to the input variations. The robust model has solid stability in its outcomes when there are a few chances in the input variables. In this study, the uncertainty analysis was performed for the best models (RELM-BWO) throughout the testing phase. The values bracketed by lower and upper *95PPU* for both lakes exceeded 88.5% (see Fig 14). Notably if the value bracketed by *95PPU* is more than 80%, the models have produced good forecasting [109]. However, Fig 15 shows that the *d-factor* values generally have a gradual increase when the lead time increases. For Lake Superior, the model provides excellent forecasting for the one-and two-month step-ahead drought because the forecasting results have less uncertainty with *d-factor* values ranging from 0.493 to 0.595. Conversely, the model provides a higher uncertainty (*d-factor* is more than one) in three-, four-, five, and six-month ahead, indicating that the drought forecasting results in these time intervals are not good, and the model performs poorly. According to the same Figure, it can be observed that the forecasting accuracy of Lake Michigan-Huron is slightly better than Lake Superior because it provides a lower value of the *d-factor*, supporting outcomes of previously described statistical indicators. The slight discrepancy in the accuracy of drought prediction may be due to the different hydrological characteristics of the two studied lakes. Other factors, such as the volume, surface area of the studied lakes, and regulation operations, may affect the fluctuation of the levels of those lakes and consequently influence the drought patterns. It can be said that the suggested model has a solid level of accuracy when forecasting a drought of one-, two-, and three-month ahead because the *d-factor* (less than one) ranges from 0.403 to 0.885.

Forecasting hydrological drought for extended time horizons is challenging due to various factors that can increase uncertainty and errors in the forecast. As the forecasting horizon increases, the relationship between inputs and outputs becomes more complicated, hindering prediction accuracy. This increasing complexity often results in higher levels of uncertainty, making it more challenging to predict future values accurately. Other factors impacting forecast accuracy include changes in underlying data patterns, seasonal effects, and unexpected events that affect data patterns.

### 5.5 Investigating the use of RELM-BWO for forecasting drought in Lake Ontario, Lake Erie, and Lake St. Clair

It was also demonstrated that the proposed model (RELM-BWO) effectively forecasts drought conditions in two greatest lakes. Therefore, it is crucial to utilize this model for forecasting droughts in the remaining three lakes (Lake Ontario, Lake Erie, and Lake St. Clair) that have not experienced severe droughts in the past fifty years. The performance metrics of the model’s drought forecasts, ranging from one to six months in advance for all lakes, are provided in Table 5. The quantitative results indicate that the model exhibits the highest accuracy in forecasting droughts for Lake St. Clair (*MAE* ranging from 0.0184 to 0.1139, *RMSE* ranging from 0.0240 to 0.1383, and *MAPE* ranging from 5.96 to 33.75%). The model also demonstrates the lowest *AAETP* values (ranging from 0.0178 to 0.1091) and the highest *R*^*2*^ values of 0.9905 to 0.9635). The model’s accuracy is satisfactory for the other two lakes, with Lake Ontario exhibiting the least accurate forecasts compared to the other lakes due to higher uncertainty values. Uncertainty analysis shows that the model reliably forecasts droughts up to six months in advance, except for Lake Ontario, where dependable forecasts are generated only up to three months ahead.

**Table 5:** Performance indicators of the hybrid model for Lake Ontario, Lake Erie, and Lake St. Clair, the use of bold numbers indicates that the forecasting is deemed unreliable due to significant uncertainty in the projected values.

Lake/ lead time forecasting		Statistical Parameter					Uncertainty Analysis	
**Lake Ontario**	Interval	MAE	RMSE	MAPE%	R^2^	AAETP	PPU95	d-factor
	t+1	0.0260	0.0327	15.31	0.9853	0.0209	85.62	0.4271
	t+2	0.0574	0.0717	24.15	0.9701	0.0347	69.23	0.59.15
	t+3	0.0896	0.1153	45.46	0.9383	0.0592	61.74	0.6545
	t+4	**0.1276**	**0.1603**	**65.74**	**0.8906**	**0.1026**	**49.33**	**0.6704**
	t+5	**0.1542**	**0.1946**	**95.46**	**0.8446**	**0.1432**	**48.32**	**0.7790**
	t+6	**0.1805**	**0.2287**	**114.47**	**0.7974**	**0.1703**	**57.38**	**1.0757**
**Lake Erie**	t+1	0.0207	0.0269	17.33	0.9891	0.0189	0.88	0.2666
	t+2	0.0400	0.0499	22.38	0.9871	0.0304	79.60	0.3746
	t+3	0.0626	0.0774	55.53	0.9830	0.0495	80.27	0.3753
	t+4	0.0849	0.1036	88.54	0.9776	0.0659	67.45	0.5009
	t+5	0.0997	0.1239	110.82	0.9719	0.0860	68.46	0.6170
	t+6	0.1265	0.1515	171.17	0.9634	0.1134	65.77	0.6624
**Lake St. Clair**	t+1	0.0184	0.0240	5.96	0.9905	0.0178	86.96	0.2524
	t+2	0.0345	0.0442	11.15	0.9884	0.0219	84.28	0.3722
	t+3	0.0537	0.0673	17.97	0.9841	0.0421	72.48	0.4156
	t+4	0.0716	0.0902	23.54	0.9784	0.0758	68.12	0.4491
	t+5	0.0929	0.1146	28.31	0.9723	0.0963	64.77	0.4667
	t+6	0.1139	0.1383	33.75	0.9635	0.1091	64.09	0.5195

### 5.6 Discussion

The study’s results indicate that including the applied metaheuristic algorithm (BWO) significantly improves the accuracy of classical approaches like RF, SVR, and ELM in drought forecasting across different lead times. These results are consistent with previous research [51, 110], indicating that employing hybridized machine learning methods improves forecast accuracy and overall predictive performance. By utilizing the BWO algorithm to optimize hyperparameters of standalone models, the forecasting of MSWI in the studied lakes becomes more accurate. Both statistical indices and visual inspections confirm that the BWO algorithm significantly enhances the accuracy of all models when forecasting hydrological drought based on MSWI. The BWO algorithm provides precise parameters to the models, resulting in an increase in their generalization capacity. The RELM-BWO model emerges as the most effective among the hybrid models tested. The study illustrates that the optimal computation of the regularization parameter using the BWO algorithm has made the RELM-BWO model superior to the ELM-BWO model. By enhancing accuracy and performance, the BWO algorithm solidifies the RELM-BWO model as the top-performing hybrid model for MSWI-based drought forecasting.

As the forecasting lead time increases, the accuracy of all hybrid models gradually decreases, making it challenging to select reliable forecasts. To address this, uncertainty analysis proves to be an effective tool for identifying reliable forecasts. The study reveals that some lakes can reliably forecast drought up to 2 months ahead, while others require 3- or 6-months lead time. The varying accuracy may be attributed to the data quality, indicating that models trained with different drought conditions yield more accurate forecasting results.

## 6. Conclusion

This study utilized four hybrid ML-based models (SVR-BWO, ELM-BWO, RF-BWO, and RELM-BWO) and three classical models (ELM, RF, and SVR) to forecast drought in the Great Lakes region for several months in advance (1 to 6 months). Drought calculations were performed via MSWI using water level data from 1918 to 2020. The dataset was split into a training set (December 1921 to January 1997) and a testing set (February 1997 to December 2021) for model development. Over the past 50 years, severe drought events have been limited to Lake Superior and Lake Michigan-Huron. Thus, the selected models were employed to forecast droughts for several months in advance, specifically for these two lakes, and the best model was used to forecast the drought for the other three lakes. To assess the models’ ability to capture turning points, a new indicator called *AAETP* was introduced, as traditional criteria (*RMSE* and *R*^*2*^) were sometimes insufficient due to high auto-correlation in the drought dataset. The hybrid models, with BWO optimization, outperformed the classical models. Among them, RELM-BWO was the most accurate and reliable model. Evaluating the recommended model’s effectiveness in reducing *AAETP* for various lead times, it was observed that, in the case of Lake Superior, it achieved substantial improvements of 37.33%, 76.74%, and 32.21% compared to SVR, RF, and ELM, respectively. Similarly, for Lake Michigan-Huron, the RELM-BWO model demonstrated reductions of 16.18%, 56.27%, and 22.93% in *AAETP* compared to SVR, RF, and ELM, respectively. Also, the RELM-BWO consistently outperformed other hybrid models, with enhancements of 17.27% to 54.69% for Lake Superior and 7.21% to 39.35% for Lake Michigan-Huron.

Generally, as the forecast lead time increased, all models’ accuracy declined. However, the proposed model showed a slightly smaller accuracy deterioration than the others. To ensure the reliability of the forecasting results, uncertainty analysis was conducted using *d-factor* and *95PPU* criteria. The analysis exhibited that RELM-BWO reliably forecasts droughts up to two months in advance for Lake Superior and up to three months ahead for Lake Michigan-Huron. Furthermore, uncertainty analysis confirmed that RELM-BWO can estimate droughts up to six months in advance for Lake Erie and Lake St. Clair, and up to three months ahead for Lake Ontario.

## Supporting information

S1 Appendix (DOCX)

S1 File Water level data. (CSV)

S2 File Droughts. (XLSX)
