# Supplementary material for: Machine learning models development for accurate multi-months ahead drought forecasting: Case study of the Great Lakes, North America
Source: PLoS One. 2023 Oct 31;18(10):e0290891. doi: 10.1371/journal.pone.0290891 (PMC10617742; doi:10.1371/journal.pone.0290891)
Supplement: S1 Appendix — (DOCX) [file pone.0290891.s001.docx]

## Appendix A: Figures


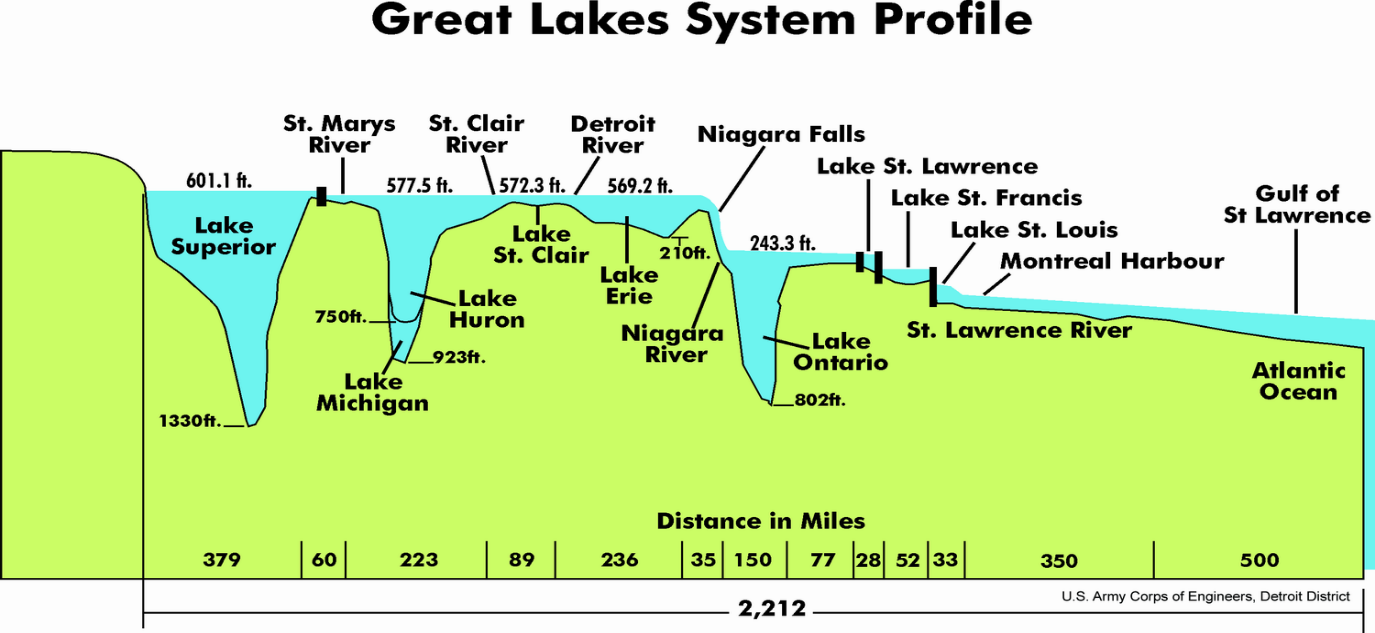


A1 Fig. Profile of the Great Lakes system (source: https://commons.wikimedia.org/wiki/File:Great_Lakes_2.PNG)

A2 Fig. drought and water level over time for Lake Erie. a) Multivariate water level index (MWI), b) Water level elevation.

A3 Fig. Drought and water level over time for Lake Ontario. a) Multivariate water level index (MWI), b) Water level elevation.

A4 Fig. Drought and water level over time for Lake St. Clair. a) Multivariate water level index (MWI), b) Water level elevation.
